# Metatranscriptomic profiling reveals diverse tick‐borne bacteria, protozoans and viruses in ticks and wildlife from Australia

**DOI:** 10.1111/tbed.14581

**Published:** 2022-05-16

**Authors:** Alexander W. Gofton, Kim R. Blasdell, Casey Taylor, Peter B. Banks, Michelle Michie, Emilie Roy‐Dufresne, Jacqueline Poldy, Jian Wang, Michael Dunn, Mary Tachedjian, Ina Smith

**Affiliations:** ^1^ CSIRO Health and Biosecurity Canberra, Connecticut Australia; ^2^ CSIRO Health and Biosecurity Australian Centre for Disease Preparedness Geelong VIC Australia; ^3^ School of Life and Environmental Sciences University of Sydney Sydney NSW Australia

**Keywords:** *Borrelia*, metagenomics, metatranscriptomics, tick‐borne pathogens, ticks, zoonoses

## Abstract

Tick‐borne zoonoses are emerging globally due to changes in climate and land use. While the zoonotic threats associated with ticks are well studied elsewhere, in Australia, the diversity of potentially zoonotic agents carried by ticks and their significance to human and animal health is not sufficiently understood. To this end, we used untargeted metatranscriptomics to audit the prokaryotic, eukaryotic and viral biomes of questing ticks and wildlife blood samples from two urban and rural sites in New South Wales, Australia. *Ixodes holocyclus* and *Haemaphysalis bancrofti* were the main tick species collected, and blood samples from *Rattus rattus, Rattus fuscipes, Perameles nasuta* and *Trichosurus vulpecula* were also collected and screened for tick‐borne microorganisms using metatranscriptomics followed by conventional targeted PCR to identify important microbial taxa to the species level. Our analyses identified 32 unique tick‐borne taxa, including 10 novel putative species. Overall, a wide range of tick‐borne microorganisms were found in questing ticks including haemoprotozoa such as *Babesia*, *Theileria, Hepatozoon* and *Trypanosoma* spp., bacteria such as *Borrelia*, *Rickettsia*, *Ehrlichia*, *Neoehrlichia* and *Anaplasma* spp., and numerous viral taxa including *Reoviridiae* (including two *coltiviruses*) and a novel *Flaviviridae*‐like *jingmenvirus*. Of note, a novel hard tick‐borne relapsing fever *Borrelia* sp. was identified in questing *H. bancrofti* ticks which is closely related to, but distinct from, cervid‐associated *Borrelia* spp. found throughout Asia. Notably, all tick‐borne microorganisms were phylogenetically unique compared to their relatives found outside Australia, and no foreign tick‐borne human pathogens such as *Borrelia burgdorferi s.l*. or *Babesia microti* were found. This work adds to the growing literature demonstrating that Australian ticks harbour a unique and endemic microbial fauna, including potentially zoonotic agents which should be further studied to determine their relative risk to human and animal health.

## 1 INTRODUCTION

Ticks are important disease vectors and can transmit a diverse array of infectious agents to their vertebrate hosts, including bacteria, protozoa, helminths and viruses (de la Fuente et al., 2008). Incidences of human tick‐borne disease are increasing in many parts of the world due to climate change and changes in land use (Diuk‐Wasser et al., 2020; Gilbert, 2021). While the risks posed by ticks as vectors of zoonotic disease are well studied in parts of Europe and North America, in Australia, the significance of ticks as vectors of emerging zoonoses is not well understood.

Australia has a unique tick fauna, most of which are endemic only to Australia and New Guinea and are evolutionarily distinct to their relatives elsewhere (Barker & Walker, 2014; Roberts, 1970). For example, Australian *Ixodes*, *Amblyomma* and *Bothriocroton* species ticks form deeply rooted monophyletic lineages that diverged from their overseas relatives 112–51 mya (Beati & Klompen, 2019; Charrier et al., 2018). Although Australia's tick fauna is unique and diverse, few species are anthropophilic, with *Ixodes holocyclus*, *Ixodes tasmani* and *Haemaphysalis bancrofti* being the most common in eastern Australia (Barker & Walker, 2014; Kwak, 2018).

The emergence of tick‐borne zoonoses is of longstanding community concern in parts of Australia, particularly in relation to a poorly described chronic illness that is historically associated with tick bites (Chalada et al., 2016; Collignon et al., 2016). While the association to tick‐bites is predominantly anecdotal (Brown, 2018; Storey‐Lewis, 2019), this illness has been termed Debilitating Symptom Complexes Attributed to Ticks (DSCATT) to acknowledge the multifaceted nature of the illness and to create clarity from other terminology such as ‘Lyme‐like disease’ which had previously been used. *Borrelia burgdorferi s.l*., the aetiological agent of Lyme disease, has not been reliably identified in Australian ticks, wildlife, domestic animals or people (except for nonautochthonous infections diagnosed in Australia) (Chalada et al., 2016; Collignon et al., 2016; Egan, Loh, et al., 2020; Egan, Taylor, Austen, et al., 2021; Gofton et al., 2015; Harvey et al., 2019; Irwin et al., 2017). Indeed, compared to other continents, Australia has relatively few tick‐borne human pathogens, including several endemic *Rickettsia* spp. (*R. australis, R. honei* and *R. honei* subsp*. marmionii*) (Dehhaghi et al., 2019). Other facultative tick‐borne pathogens such as *Coxiella burnetti*, *Burkholderia pseudomallei, Francisella tularensis holarctica* and *F. hispaniensis* also occur in Australia; however, the relative importance of tick‐borne transmission in the epidemiology of these pathogens is thought be minimal (or is undocumented) compared to other transmission routes (Dehhaghi et al., 2019).

Despite the relative paucity of tick‐borne human pathogens in Australia, there appears to be a rich diversity of microorganisms carried by Australian ticks, and there is a growing body of work demonstrating that Australian ticks and wildlife harbour unique microbial fauna, including some potentially zoonotic agents. For example, recent studies have identified novel *Borrelia* (Beard et al., 2021; Loh et al., 2016; Panetta et al., 2017), *Ehrlichia* (Gofton et al., 2017, 2018), *Neoehrlichia* (Egan, Loh, et al., 2020; Gofton et al., 2016), *Babesia* (Greay et al., 2018; Loh et al., 2018; Storey‐Lewis et al., 2018), *Iflaviridae* (O'Brien et al., 2018) and *Reoviridae* (Harvey et al., 2019) in Australian ticks and wildlife. However, it should be noted that studies seeking to clarify the zoonotic potential of these microorganisms are currently lacking.

Metagenomics has emerged as a powerful tool for disease surveillance due to the technology's ability to identify taxonomically diverse and uncharacterised microorganisms in a wide range of sample types without a priori hypotheses. While metatranscriptomics has primarily been used for functional microbiome profiling or for the detection and characterisation of viruses, it has the power to detect and classify RNA molecules from all domains of life (Batson et al., 2021; Larsen et al., 2016; Marcelino et al., 2019; Ortiz‐Baez et al., 2020; Westreich et al., 2019). To this end, we utilised metatranscriptomic sequencing to simultaneously investigate the prokaryotic, eukaryotic, and viral biomes of questing ticks and wildlife blood samples with the aim of identifying novel potentially zoonotic tick‐borne microorganisms at two study sites in coastal New South Wales (NSW), Australia.

## 2 MATERIALS AND METHODS

### 2.1 Tick and wildlife sampling

**FIGURE 1:**
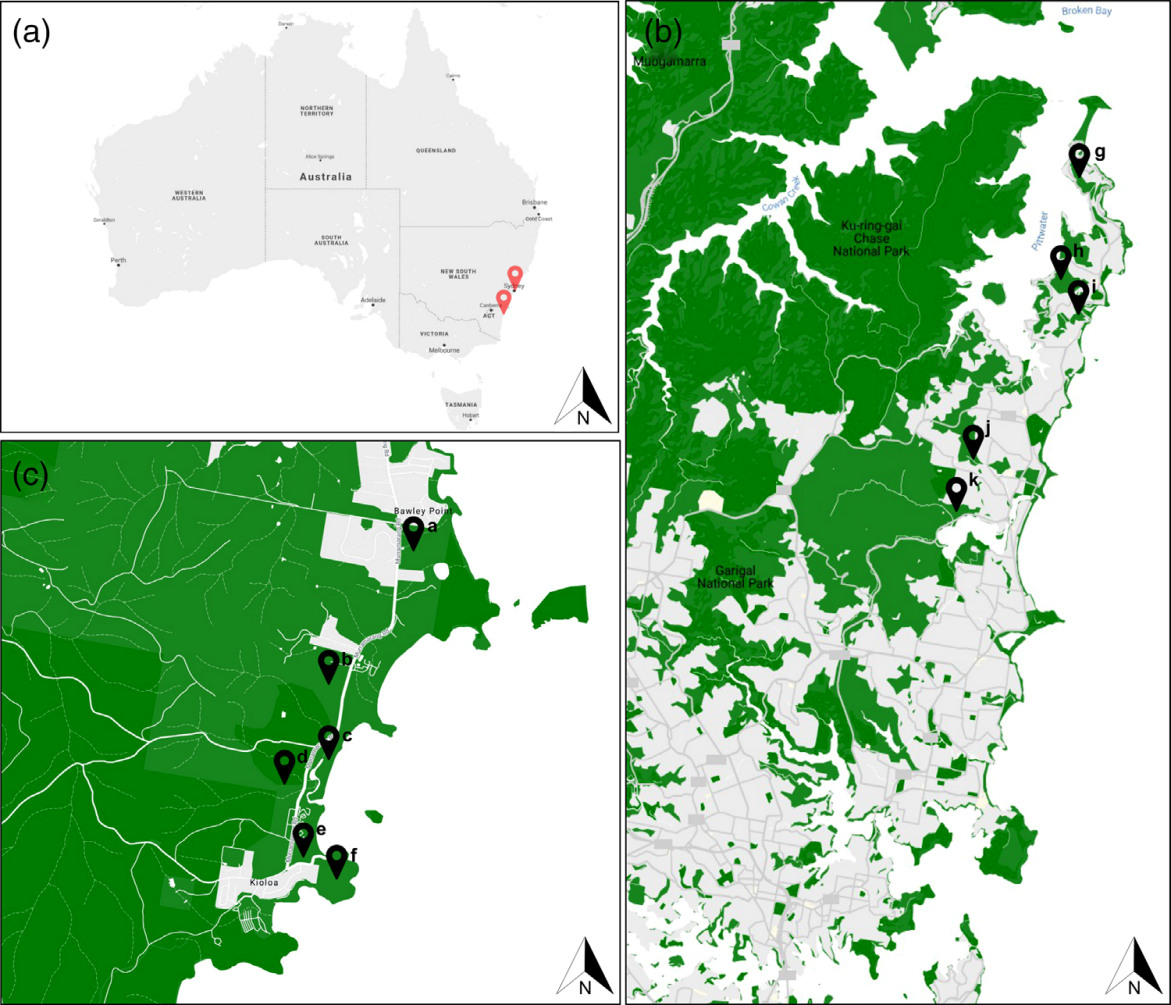
Map of sampling sites. (a) Map of Australia showing geographic context of the two sampling locations (red icons). (b) Inset map of northern Sydney, NSW, showing sampling sites in remnant urban bushland. (c) Inset map of Kioloa, NSW, showing sampling sites in native bushland. Green layers in (b) and (c) indicate natural vegetation, grey indicates urban areas. Details of specific sampling locations are presented in the Supporting Information

Ticks and wildlife samples were collected from October 2019 to December 2020 from two study areas, one located on the Northern Beaches area of northern Sydney, (NSW), Australia, and one located in the township of Kioloa, on the mid‐south coast of NSW, Australia (Figure 1). Sites were in remnant urban bushland or urban‐adjacent rural bushland. At each sampling event, questing ticks were collected over a 3‐day period by dragging a 1 m^2^ white flannel cloth across leaf litter and over low‐lying vegetation (Newman et al., 2019; Simmons et al., 2021) and removing tick with forceps into vials. Questing ticks were frozen and stored in the field on dry ice before being stored at −80°C within 3 days of collection. Ticks were morphologically identified to species, life stage, and sex using standard keys (Barker & Walker, 2014; Roberts, 1970). During sampling, which was impeded by bushfires and SARS‐CoV‐2 travel restrictions, efforts were made to maximise the number of samples obtained; however, samples were not collected in a systematic manner that allowed the calculation of tick density.

Ground dwelling small‐ to medium‐sized mammals were targeted from September to December 2020 in Elliot‐style (46 × 15.5 × 15 cm) and cage‐style (72 × 32 × 31 cm) traps set over three consecutive nights and baited with peanut butter and oats balls. Native wildlife (*Rattus fuscipes*, *Perameles nasuta* and *Trichosurus vulpecula*) were lightly anesthetised with 1–5% vaporised isoflurane and venous blood collected prior to recovery and release. Exotic rodents (*Rattus rattus*) were humanely killed, and blood was collected from the heart into EDTA tubes during necropsy. Blood samples were frozen in the field in dry ice and stored at −80°C within 3 days of collection. Tick and wildlife sampling was performed as approved by the CSIRO Animal Ethics Committee (approval no. 2020‐04) and NSW Department of Planning, Industry and Environment Scientific Licences SL102309 and SL102426.

### 2.2 Nucleic acid extraction

Ticks (*n *= 3665) were externally decontaminated with 5% sodium hypochlorite for 3 min, washed in 70% ethanol and air dried (Hoffmann et al., 2020). Ticks were then pooled according to species, life stage/sex and collection site (Table 1). Uneven tick pool sizes were utilised in order to maximise the total number of ticks included in each sequencing run at the expense of statistical precision in estimating the prevalence of tick‐borne microorganism and having a standardised dilution factor across pooled samples.

Tick pools were homogenised in Lysing Matrix I or H tubes (MP Biomedicals, USA) with 300 μl of DNA/RNA Shield™ (Zymo Research, USA) using a Precellys® 24 Tissue Homogenizer (Bertin Instruments, France) by bead‐beating at 6000 Htz for 1 min at 4°C. Supernatant was separated by centrifugation (14,000 rcf for 3 min) and 200 μl used for DNA and RNA parallel purification using the *Quick*‐DNA/RNA™ MagBead Kit (Zymo Research, USA) following the manufacturer's protocol. All wildlife blood samples were lysed in an equal volume of 2× DNA/RNA shield™ and RNA extracted using the Zymo *Quick*‐DNA/RNA™ MagBead Kit following the manufacturer's protocol. DNA was extracted from blood samples using the Qiagen DNeasy Blood and Tissue Kit (QIAGEN, Germany) following the manufacturer's protocol. RNA quality and quantity were assessed using the Agilent 2100 BioAnalyzer with the RNA 6000 Nano Kit (Agilent Technologies, USA), and DNA quantity was assessed with the Qubit™ 3.0 fluorometer dsDNA BR Assay Kit (Invitrogen™, USA). Nucleic acid samples were stored at −80°C until use. To achieve sufficient RNA concentrations for downstream applications some RNA samples were concentrated with the Zymo RNA Clean & Concentrator™ Kit (Zymo Research, USA). Prior to nucleic acid extraction, ticks underwent one freeze–thaw–freeze cycle while being morphologically identified and pooled, while blood samples were only thawed once immediately prior to extraction.

### 2.3 High‐throughput sequencing

Metatranscriptomic sequencing libraries were generated from up to 1 μg of RNA using the Zymo‐Seq RiboFree™ Total RNA Library Kit (Zymo Research, USA), following the manufacturer's protocol, except that AMPure XT beads (Beckman Coulter, USA) were used in place of Select‐a‐Size MagBeads. Libraries were dual indexed with the Zymo‐Seq™ UDI Primer Plate Kit (Zymo Research, USA) following the manufacturer's protocol, purified twice using 0.8–1.2 volumes of AMPure XT beads and quantified using the Agilent 2200 TapeStation with D1000 HS screen tapes (Agilent Technologies, USA). Libraries were sequenced on the Illumina® NovaSeq™ 6000 platform (Illumina Inc., USA) using 300 cycle paired‐end chemistry.

### 2.4 Metatranscriptomic analysis

Raw sequences were trimmed and quality‐filtered using cutadapt v2.8 (Martin, 2011), trimmomatic v0.38 (Bolger et al., 2014) and *dedupe* (implemented in BBTools v38.37). Quality‐filtered reads were assembled using Trinity v2.11.0 (Grabherr et al., 2011). Contig abundance were normalised using transcripts per million (TPM) (Bo & Dewey, 2011), calculated by mapping reads back to the assembled contigs using bowtie2 v2.3.4 (Langmead & Salzberg, 2012). Raw Illumina data have been accessioned in the NCBI Sequence Read Archive (PRJNA777641).

Assembled transcripts were aligned to GenBank's nt and nr databases (downloaded 13 November 2020) using BLASTn (*megablast*) v2.11.0 (Altschul et al., 1990) and DIAMOND (*blastx*) v0.9.28 (Buchfink et al., 2015), respectively. Hierarchical taxonomic assignments were produced by calculating the lowest common ancestor (LCA) from BLASTn or DIAMOND hits using the weighted LCA algorithm implemented in MEGAN v6.20.19 (Huson et al., 2016). BLASTn LCA taxonomic classifications were used to classify Eukaryotic and Prokaryotic contigs because many taxa are classified solely by ribosomal RNA (rRNA), while DIAMOND LCA taxonomies were used to assign viral taxa. For tick and wildlife samples, host contigs were defined as those with an LCA taxonomy assigned to the Order Ixodidae or Class Mammalia (or lower), respectively, and were excluded, as were plant and fungal taxonomies and taxonomies that failed to resolve to at least the Phylum level.

LCA taxonomies and TPM‐normalised abundances were used to analyse microbial community composition in R v4.0.3 utilising ampviz2 v2.7.9 (Andersen et al., 2018), phyloseq v1.34 (McMurdie & Holmes, 2013) and microbiomeutilities v0.99.02. packages. Alpha diversity was calculated using Chao1 and Shannon indices with statistical comparisons performed using pairwise Wilcoxon's nonparametric tests. Principal coordinate analysis using the Jensen‐Shannon Divergence was used to compare microbial beta diversity between sample species and tick life stages. Taxonomic heatmaps were produced by summarising taxonomies at the family level (or higher) with read counts transformed into relative abundances per sample.

### 2.5 PCR confirmation of novel tick‐associated microorganisms

PCR and qPCR assays were used to confirm the detection of selected novel microorganisms and screen for known tick‐borne pathogens in all pooled tick samples and individual wildlife blood samples. PCR primers, probes and assay conditions are provided in Table S1. PCR products were electrophoresed through 1–2% agarose gels and positive PCR products excised, purified and Sanger sequenced using both 5′ and 3′ PCR primers. Forward and reverse chromatograms were aligned with MAFFT (Katoh et al., 2002) in Geneious Prime v2020.2.5 (Kearse et al., 2012) before being aligned to reference sequences with MAFFT. Maximum likelihood phylogenetic analyses were performed in IQ‐TREE (Minh et al., 2020) with model selection (Kalyaanamoorthy et al., 2017) and 5000 bootstrap replicates (Hoang et al., 2018). The prevalence of tick‐borne microorganisms in variable‐sized tick pools was calculated using maximum likelihood estimation according to Williams and Moffitt (2001).

## 3 RESULTS

### 3.1 Sample collection

A total of 3665 questing ticks were collected from field sites (Figure 1), morphologically identified as either *I. holocyclus*, *H. bancrofti* or *I. trichosuri* and pooled into groups according to collection site, species, life stage and sex for nucleic acid extraction (Table 1). A total of 3125 ticks were collected from Kioloa and grouped into 130 pools, including 99 and 31 *I. holocyclus* and *H. bancrofti* pools, respectively, containing a total of 2215 and 910 ticks, respectively. A total of 505 *I. holocyclus* ticks were collected from northern Sydney and grouped into 48 pools along with 30 *I. trichosuri* nymphs and five *H. bancrofti* females, grouped into one pool each (Table 1).

**TABLE 1:** Summary of ticks collected and pooled

Collection events (1 per month listed)	Species	Life stage	Number collected	Number of pools	Pool sizes (# ticks, # pools)
Kioloa, NSW					
October 2019	*I. holocyclus*	Nymph	400	13	(50, 2) (30, 8) (25, 2) (10, 1)
		Male	35	4	(10, 3) (5, 1)
		Female	15	2	(10, 1) (5, 1)
	*H. bancrofti*	Nymph	195	6	(50, 1) (30, 4) (25, 1)
		Male	5	1	(5, 1)
November 2019	*I. holocyclus*	Nymph	300	10	(30, 10)
		Male	65	11	(10, 2) (5, 9)
		Female	50	8	(10, 2) (5, 6)
	*H. bancrofti*	Nymph	90	3	(30, 3)
		Male	5	1	(5, 1)
		Female	5	1	(5, 1)
June 2020	*I. holocyclus*	Nymph	600	20	(30, 20)
October 2020	*I. holocyclus*	Nymph	550	11	(50, 11)
		Male	60	6	(10, 6)
		Female	50	5	(10, 5)
	*H. bancrofti*	Nymph	500	10	(50, 10)
		Male	10	2	(5, 2)
		Female	10	2	(5, 2)
December 2020	*I. holocyclus*	Male	40	4	(10, 4)
		Female	50	5	(10, 5)
	*H. bancrofti*	Nymph	50	1	(50, 1)
		Male	20	2	(10, 2)
		Female	20	2	(10, 2)
		Total	3125	130	
Barrenjoey Peninsula, Sydney					
November 2019	*I. holocyclus*	Nymph	90	3	(30, 3)
		Male	40	8	(5, 8)
		Female	40	8	(5, 8)
December 2019	*I. holocyclus*	Male	15	3	(5, 3)
		Female	15	3	(5, 3)
August 2020	*I. holocyclus*	Nymph	150	5	(30, 5)
	*I. trichosuri*	Nymph	30	1	(30, 1)
September 2020	*I. holocyclus*	Nymph	90	3	(30, 3)
		Female	5	1	(5, 1)
November 2020	*I. holocyclus*	Male	30	6	(5, 6)
		Female	30	6	(5, 6)
	*H. bancrofti*	Female	5	1	(5, 1)
		Total	540	48	
		Total	3665	187	

Blood samples (*n *= 69) were collected from 33 black rats (*Rattus rattus*), 11 bush rats (*Rattus fuscipes*), 19 long‐nosed bandicoots (*Perameles nasuta*) and six brush‐tailed possums (*Trichosurus vulpecula*). Urban northern Sydney field sites were dominated by *R. rattus* (*n *= 33), compared to the more rural landscape of Kioloa where native *R. fuscipes* were the dominant species trapped (*n *= 11). *Perameles nasuta* and *T. vulpecula* were trapped at both field sites; however, *P. nasuta* were more common in northern Sydney (*n *= 18) compared to Kioloa (*n *= 1), while equal numbers (*n *= 3) of *T. vulpecula* were trapped in northern Sydney and Kioloa.

### 3.2 Metatranscriptomic analysis

Metatranscriptomic sequencing was performed on 107 of 178 tick pools, and 45 of 69 wildlife blood samples where RNA quality and quantity were sufficient to produce high‐quality sequencing libraries. High throughput sequencing produced an average of 3,49,01,192 quality filtered read‐pairs per sample which generated between 14,272 and 49,96,561 de novo contigs per sample (median: 12,24,445) (Table S2). Filtering of host and unassigned contigs removed an average of 99.33% and 68.48% of contigs from wildlife blood and tick samples, respectively, leaving 2163 unique taxonomic classifications of which 1996 were assigned to at least a family‐level taxonomy.

Alpha diversity calculations showed no significant difference (*p *> .01) in microbial diversity levels between tick species, site, or between different life stages between or within species (Figures S1, S2). When grouped together, wildlife blood samples had significantly less (*p <* .01) microbial diversity compared to tick samples, but there was no significant (*p *> .01) difference in diversity levels between wildlife species, although some *R. rattus* and *T. vulpecula* samples had elevated microbial diversity compared to other samples (Figure S2).

**FIGURE 2:**
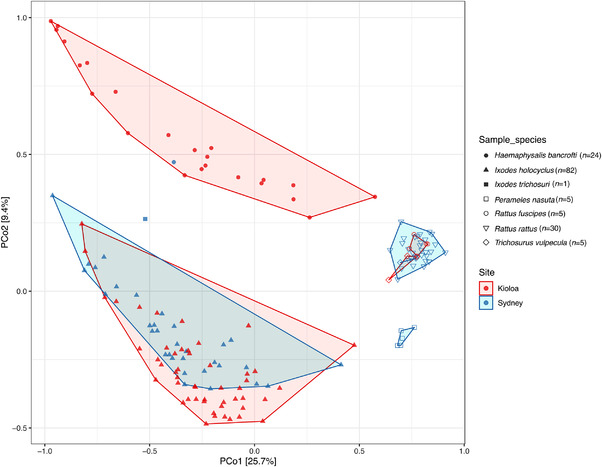
PCoA analysis of the microbial communities in tick and wildlife samples using Jensen‐Shannon Divergences. Coloured polygons frame samples of the same species and collection site

PCoA ordination of beta diversity demonstrated that tick species have distinctive microbial community profiles, with *I. holocyclus*, *H. bancrofti* and *I. trichosuri* samples forming discrete clusters (Figure 2). Within *I. holocyclus* samples, there was a clear distinction between the beta diversity profiles of nymph and adult samples, which has been demonstrated for other tick species (J. C. Gil et al., 2020) (Figure S3). However, this trend was not apparent in *H. bancrofti* samples (Figure S3). The microbial profile of wildlife samples was distinct from that of tick samples, although *R. rattus, R. fuscipes* and *T. vulpecula* samples overlapped with each other indicating a degree of shared microbial taxa (Figure 2). Conversely, *P. nasuta* samples formed a distinct cluster separate from other wildlife samples, indicating a unique microbial community profile (Figure 2).

**FIGURE 3:**
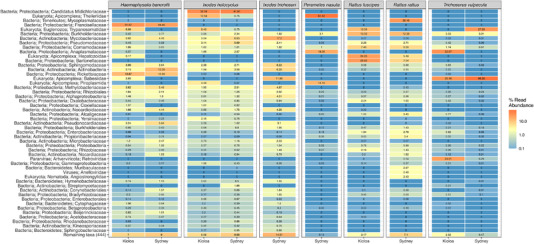
Heatmap of the relative abundance of the top 50 microbial taxa identified by metatranscriptomic analysis and summarised at the family level (or higher). Values represent the median relative abundance of TPM‐adjusted contig counts

As expected, the microbial fauna of tick samples was dominated by bacterial endosymbionts, with *Candidatus* Midichloria sp. comprising a median relative abundance (MRA) of 38.41% of microbial reads in *I. holocyclus* samples, and *Francisella* sp. and *Rickettsia* sp. endosymbionts comprising a MRA of 31.27% and 13.73%, respectively, in *H. bancrofti* samples (Figures 3, S4, S6) (Beninati et al., 2009; Greay et al., 2021). No dominant bacterial endosymbiont was identified in the single *I. trichosuri* pool, although Mycobacteriaceae were highly abundant (MRA: 17.2%).

A number of vertebrate‐infecting tick‐borne organisms and other potentially zoonotic organisms were also identified in tick and wildlife samples, including Anaplasmataceae, Rickettsiaceae, Bartonellaceae, Borreliaceae, Babesiidae, Theileriidae, Hepatozoidae, Trypanosomatidae and various arboviral families (Table 2). The remaining abundant taxonomic diversity detected in tick and wildlife samples is summarised in Figure 3.

### 3.3 Tick‐borne Rickettsiales bacteria

In addition to endosymbiotic *Rickettsia* sp. found in *H. bancrofti*, *Rickettsia* contigs were also present in four *I. holocyclus* samples (MRA: 0.04%), including two nymph pools and one female pool from Kioloa, and one nymph pool from northern Sydney (Table 2). qPCR and sequencing of *gltA* and *ompB* amplicons confirmed these contigs belonged to *R. australis*, with > 99.6% sequence similarity to the *R. australis* str. Cutlack reference genome (GenBank: CP003338).

**FIGURE 4:**
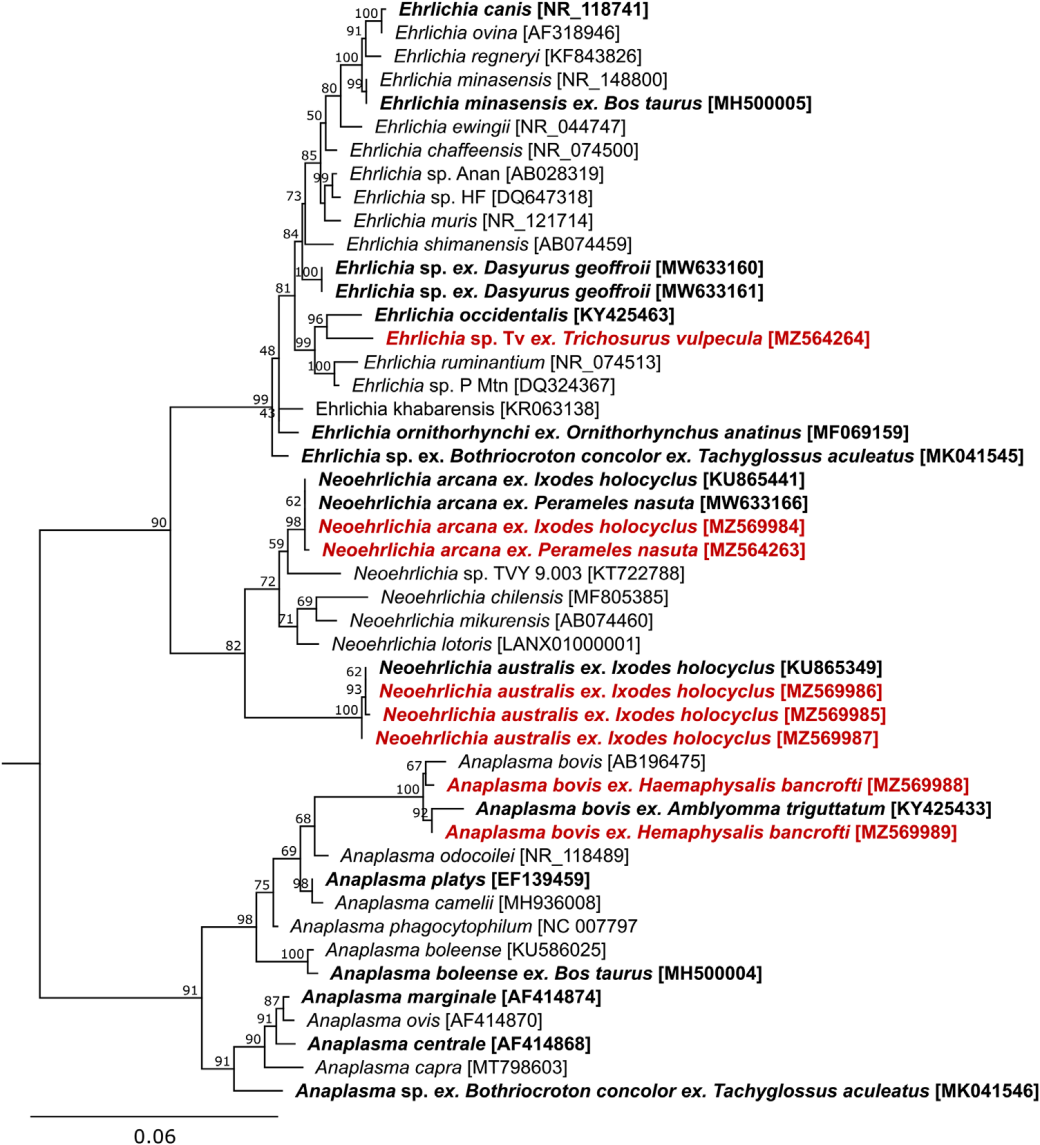
Maximum likelihood phylogenetic analysis of Anaplasmataceae 16S rRNA sequences (1200 bb). Red bold text indicates sequences from this study, bold text indicates Anaplasmataceae species previously identified in Australia. Square brackets indicate GenBank accessions. Tree rooted with *Rickettsia rickettsii* [NR_028018], not shown

Anaplasmataceae contigs were identified in 29 and 20 *I. holocyclus* samples from Kioloa and northern Sydney, respectively, and were more abundant in nymph samples across both field sites, and overall, more prevalent and abundant in samples from northern Sydney (MRA: 2.57%) compared to Kioloa (MRA: 1.66%) (Figure S4, Table 2). Sequencing of 16S rRNA (16S) amplicons confirmed the Anaplasmataceae species in *I. holocyclus* were *Candidatus* Neoehrlichia australis and *Candidatus* Neoehrlichia arcana (Table 2, Figure 4). *Candidatus* Neoehrlichia arcana was more prevalent in *I. holocyclus* from northern Sydney (37.9%, 95% confidence intervals [95%CI] = 21.3–61.7%) compared to Kioloa (13.2%, 95%CI = 8.5–19.1%), as was *Candidatus* Neoehrlichia australis (northern Sydney: 28.4%, 95%CI = 15.3–47.6%; Kioloa: 16.4%, 95%CI = 11.1–23.1%) (Table 2). Anaplasmataceae contigs were also identified in three *H. bancrofti* samples from Kioloa (3.6%, 95%CI = 0.9–9.3%) although at low abundance (MRA: 0.37%) and were confirmed by PCR to belong to *Anaplasma bovis* (Table 2, Figure 4).

**TABLE 2:** Summary of putative tick‐borne microorganisms and other microorganisms detected via metatranscriptomic sequencing and PCR

			Positive samples (estimated prevalence, 95% confidence intervals, minimum infection rate)	
	Host	GenBank accessions	Kioloa	Sydney
Bacterial endosymbionts				
*Francisella* sp.	*H. bancrofti*	–	31/31 (99.29%, 3.4–100%, 3.4%)	1/1 (100%, NA, 20%)
*Rickettsia* sp.	*H. bancrofti*	OK324039, OK324044	31/31 (99.29%, 3.4–100%, 3.4%)	1/1 (100%, NA, 20%)
*Ca*. Midichloria sp.	*I. holocyclus*	–	99/99 (99.38%, 5.2–100%, 4.4%)	46/46 (99.48%, 3.7–100%, 9.5%)
Rickettsiales				
*Rickettsia australis*	*I. holocyclus*	OK324035‐8, OK324040‐3	3/99 (1.4%, 0.3–3.5%,0.01% )	1/46 (2.1%, 0.1–8.9%, 0.19%)
*Ca*. Neoehrlichia australis	*I. holocyclus*	MZ569985‐7	29/99 (16.4%, 11.1–23.1%, 1.31%)	12/46 (28.4%, 15.3–47.6%, 2.3%)
*Ca*. Neoehrlichia arcana	*I. holocyclus*	MZ569984	24/99 (13.2%, 8.5–19.1%, 1.08%)	14/46 (37.9%, 21.3–61.7%, 2.77%)
	*P. nasuta*	MZ564262‐3		4/18 (22.2%)
*Anaplasma bovis*	*H. bancrofti*	MZ569988‐9	3/31 (3.6%, 0.9–9.3%, 0.32%)	
*Ehrlichia* sp. nov^a^	*T. vulpecula*	MZ564264	1/3 (33.3%)	
*Bartonella* sp.				
*Bartonella rattaustraliani*	*R. rattus*	MZ570392	–	1/33 (3.1%)
*Bartonella queenslandensis*	*R. fuscipes*	MZ570393‐4, MZ570397	5/11 (45.4%)	
*Bartonella coopersplainsensis*	*R. rattus*	MZ570396		5/33 (15.2)
	*R. fuscipes*	MZ570395	1/1 (19.1%)	
*Borrelia* sp.				
*Borrelia* sp. nov. HB^a^	*H. bancrofti*	MZ564265	2/31 (2.3%, 0.4–7.2%, 0.21%)	
Apicomplexa				
*Babesia mackerrasorum*	*H. bancrofti*	MZ502169	21/31 (42.1%, 25.8–64.9%, 2.3%)	1/1 (100%, NA, 20%)
*Babesia lohae*	*I. trichosuri*	MZ502167		1/1 (100%, NA, 3.33%)
	*T. vulpecula*	MZ502168	1/3 (33.3%)	3/3 (100%)
*Theileria* sp. cf. AU‐1048	*I. holocyclus*	MZ502183‐8	22/99 (13.12%, 8.4–19.1%, 0.99%)	
*Theileria* sp. IH^a^	*I. holocyclus*	MZ502190‐1	3/99 (1.5%, 0.4–3.9%, 0.13%)	
*Theileria* cf. peramelis	*P. nasuta*	MZ502189		10/11 (90.9%)
	*I. holocyclus*	OK037536‐8		3/46 (6.5%, 1.6–16.9%, 0.59%)
*Hepatozoon ewingi*	*H. bancrofti*	MZ502170‐2	7/31 (8.5%, 3.7%−16.5%, 7.6%)	
*Hepatozoon* sp. HB^a^	*H. bancrofti*	MZ502177‐81	10/31 (14.4%, 7.2–25.3%, 1.09%)	
*Hepatozoon* sp. R1	*R. fuscipes*	MZ502173	3/11 (36.7%)	
	*R. rattus*	MZ502175		1/33 (3.0%)
*Hepatozoon* sp. R2	*R. fuscipes*	MZ502174	1/11 (9.1%)	
	*R. rattus*	MZ502176		1/33 (3.0%)
Trypanosomes				
*Trypanosoma gilletti*	*I. holocyclus*	MZ502193‐205	19/99 (9.2%, 5.6–14.1%, 0.85%)	3/46 (6.3%, 1.6–16.4%, 0.59%)
*Trypanosoma vegrandis*	*H. bancrofti*	MZ502214	4/31 (4.8%, 1.5–11.2%, 0.43%)	
*Trypanosoma* sp. nov. HB^a^	*H. bancrofti*	MZ502209‐13	10/31 (12.4%, 6.2–21.7%, 1.09%)	
*Trypanosoma* cf. *lewisi*	*R. fuscipes*	MZ502206‐8	5/11 (45.5%)	
*Trypanosoma* cf. *cyclops*	*T. vulpecula*	MZ502192		1/3 (33.3%)
Viruses				
North Shore virus (Partitiviridae)	*I. holocyclus*	OL452116‐37	44/99 (26.8%, 19.6–35.6%, 1.98%)	26/46 (76.2%, 50.2–87.4%, 5.14%)
Shelly Headland virus (Reoviridae)	*I. holocyclus*	OL452144‐234	5/99 (2.3%, 0.8–5.1%, 0.22%)	9/46 (18.5%, 8.9–33.1%, 1.78%)
Shelly Beach virus (Reoviridae)	*I. holocyclus*	OL452138‐43		1/46 (2.1%, 0.4–8.7%, 0.19%)
Timbillica virus (Phenuiviridae)	*I. holocyclus*	OL452235‐43	38/99 (22.1%, 15.8–29.9%, 1.71%)	31/46 (79.8%, 73.1%−91.1%, 6.13%)
Genoa virus (Chuviridae)	*I. holocyclus*	OL452068‐75	14/99 (6.8%, 3.8–11.1%, 0.63%)	
Ingleside virus (Virgaviridae)	*I. holocyclus*	OL452076‐105		31/46 (81.4%, 70.1–94.4%, 6.13%)
*Ixodes holocyclus* Iflavirus (*Iflaviridae*)	*I. holocyclus*	OL452106‐15		2/46 (2.1%, 0.7–12.3%, 0.39%)
Newport tick virus (*Flaviviridae*)^a^	*I. holocyclus*	OK128264	41/99 (24.4%, 17.6–32.7%, 1.85%)	25/46 (65.2%, 4.3–93.9%, 4.95%)
Kioloa tick virus (*Rhabdoviridae*)^a^	*H. bancrofti*	OK128265	23/31 (44.4%, 28.1–66.9%, 2.25%)	
Scerri virus (*Iflaviridae*)^a^	*H. bancrofti*	OL452259	8/31 (10.7%, 4.9–20.1%, 0.87%)	
Butlers creek tick virus (*Chuviridae*)^a^	*H. bancrofti*	OL452244	7/31 (8.3%, 3.6–15.9%, 0.76%)	
O'hara headland virus (*Reoviridae*)^a^	*H. bancrofti*	OL452245	1/31 (1.1%, 0.4–4.8%, 0.1%)	
Kioloa rodent pestivirus (Flaviviridae)^a^	*R. fuscipes*	OL452246	1/11(%)	
Porters rodent pegivirus (Flaviviridae)^a^	*R. rattus*	OL452260		1/33 (3.0%)
Attunga orbivirus (Reoviridae)^a^	*R. rattus*	OL452247		8/33 (24.2%)
Anelloviridae spp.^a^	*R. fuscipes*	OL452252‐3	2/11 (18.2%)	
	*R. rattus*	OL452248‐51, OL452254‐58		8/33 (24.2%)

^a^ Indicates novel species identified in this study.

Four *P. nasuta* blood samples from northern Sydney contained Anaplasmataceae contigs (MRA: 18.05%) and were confirmed by PCR to belong to *Candidatus* Neoehrlichia arcana (Figure 4). Anaplasmataceae contigs also dominated one *T. vulpecula* blood sample from Kioloa (relative abundance: 96.21%) with 16S sequences <98.4% similar *Candidatus Ehrlichia occidentalis*. Designated *Ehrlichia* sp. Tv (for its putative host *T. vulpecula*), phylogenetic analysis demonstrated that *Ehrlichia* sp. Tv clusters as a sister taxon to *Candidatus* Ehrlichia occidentalis and together form a clade that include *Ehrlichia ruminantium* and *Ehrlichia* sp. Panola Mountain (Figure 4).

All pooled tick samples were screened for *E. canis* due to the recent outbreak of this pathogen in Australia and were negative (*Ehrlichiosis in Dogs*, 2020). Additionally, all samples were screened for *C. burnetti* and were negative. *Wolbachia* spp. contigs were identified in 12 *I. holocyclus* samples from Kioloa and northern Sydney, and in 8/24 *H. bancrofti* tick pools from Kioloa. *Wolbachia* contigs may be derived from *Ixodiphagous* spp. parasitoid wasps, which parasitize a range of Australian Ixodids (Doube & Heath, 1975; Tijsse‐Klasen et al., 2011).

### 3.4 A novel *Borrelia* sp. in *H. bancrofti* ticks

Metatranscriptomic sequencing identified 36 novel *Borrelia* sp. contigs (0.29% relative abundance) in one *H. bancrofti* nymph pool from Kioloa. Contigs included the complete 16S and 23S rRNAs, as well as partial transcripts from a range of chromosomal and plasmid‐encoded genes. Screening with *Borrelia*‐specific 16S and *flaB* PCR assays confirmed this result and detected the same *Borrelia* sp. in one additional *H. bancrofti* nymph pool from the same collection event that did not undergo metatranscriptomic sequencing (Table 2).

**FIGURE 5:**
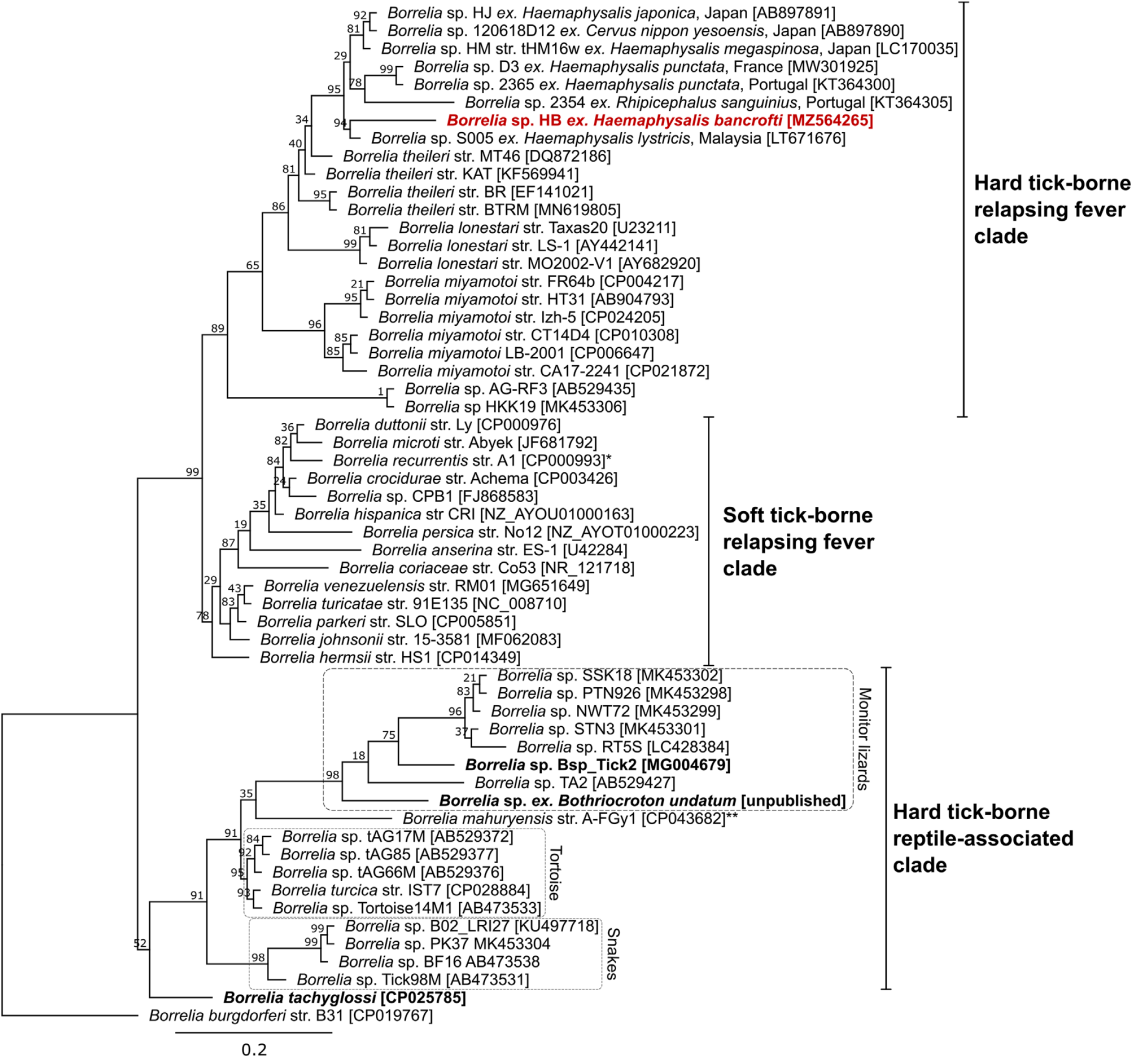
Maximum likelihood phylogenetic analysis of *Borrelia* spp. 16S rRNA sequences (1552 bp). Red bold text indicates sequences from this study, bold text indicates *Borrelia* species previously identified in Australia. Square brackets indicate GenBank accessions

*Borrelia* sp. 16S and *flaB* gene sequences were 99.26–99.92% and 94.33–98.58% similar, respectively, to novel *Borrelia* spp. associated with *Haemaphysalis* and *Rhipicephalus* ticks throughout Asia and Europe (Figures 5, S5). Phylogenetic analyses based on 16S (Figure 5) and flaB sequences (Figure S5) demonstrated that this novel *Borrelia* is distinct from closely related species in Asia and Europe and clusters within the hard tick‐borne relapsing fever clade (HTRF) which includes *B. miyamotoi*, *B. theileri* and *B. lonestari* (Nakao et al., 2021).

Additional metagenomic shotgun sequencing performed on both *Borrelia*‐positive *H. bancrofti* samples (Supporting Information) produced 211 *Borrelia* contigs totalling 45,510 bp that covered approximately 4.8% of the *Borrelia* genome. Phylogenetic analysis (Supporting Information) based on these genomic contigs concurred with 16S and *flaB* phylogenies that this novel *Borrelia* sp. is closely related to Asian *Haemaphysalis*‐associated *Borrelia* spp. but is likely a unique species with an average nucleotide identity of 94.62% to *Borrelia* sp. HM str. tHM16w across the 45,510 bp of chromosomal genome assembled (Figure S5). Herein, we refer to this species as *Borrelia* sp. HB, designated after its putative tick vector *H. bancrofti*.

### 3.5 Apicomplexan parasites in ticks and wildlife

The protozoan families Babesiidae, Theileriidae and Hepatozoidae contain only one genus each (*Babesia*, *Theileria* and *Hepatozoon*, respectively) which all contain tick‐borne vertebrate‐infecting parasites and were among the most highly abundant and prevalent taxa identified (Figure 3, Table 2). Both *Babesia* and *Hepatozoon* contigs were found in *H. bancrofti* samples from Kioloa, with *Babesia* contigs more abundance in nymph *H. bancrofti* (MRA: 3.02%) compared to male (MRA: 2.9%) or female (MRA: 0.42%) samples, while *Hepatozoon* contigs were more abundant in male *H. bancrofti* (MRA: 5.11%) compared to nymphs (MRA: 2.91%) or females (MRA: 0.52%) (Figure S6).

PCR and phylogenetic analysis of 18S rRNA (18S) sequences confirmed the presence of *Babesia* in 22/32 (43.1%, 95%CI = 25.8–64.9%) *H. bancrofti* samples and identified the species as *B. mackerrasorum* (100% similarity) (Greay et al., 2018). Two distinct *Hepatozoon* spp. occurred in *H. bancrofti* samples including *H*. *ewingi* in seven samples (8.5%, 95%CI = 3.7%−16.5%), and a novel species designated *Hepatozoon* sp. HB (for its putative vector *H. bancrofti*) in 10 samples (14.4%, 95%CI = 7.2–25.3%). *Hepatozoon* sp. HB was closely related to *H. banethi* (96.73–98.55% 18S similarity) which has only be found in Tasmania (Greay et al., 2018; Vilcins et al., 2009), and phylogenetic analyses indicated *Hepatozoon* sp. HB is likely a related sister taxon to *H. banethi* (Figure S7).

*Babesia lohae* contigs (98.1–100% 18S similarity) were also found in the single *I. trichosuri* sample from northern Sydney (11.69% relative abundance) and in *T. vulpecula* samples from northern Sydney (3/3, 100%) and Kioloa (1/3, 33.3%) (MRA: 66.58% and 30.16%, respectively) (Figures 3, S8).

*Hepatozoon* contigs were found in 4/11 (36.36%) *R. fuscipes* samples from Kioloa (MRA: 24.05%), and 2/33 (6.06%) *R. rattus* samples from northern Sydney (MRA: 5.49%) (Figure 3). Two distinct *Hepatozoon* 18S sequences were recovered from the rats, with one putative species (R1) found in three *R. fuscipes* and one *R. rattus*, and the second putative species (R2) identified in one *R. fuscpies* and one *R. rattus* (Table 2). These putative species had > 99.49% 18S sequence similarity to, and phylogenetically clustered within, a large clade of rodent‐associated *Hepatozoon* spp. including isolates from Australia (Figure S7).

In ticks, *Theileria* contigs were found in nymph *I. holocyclus* samples from Kioloa (MRA: 18.44%) and northern Sydney (MRA: 3.48%) (Figure S4), and were also the most abundant taxa in *P. nasuta* samples (MRA: 61.42%) (Figure 3). Two *Theileria* spp. were present in *I. holocyclus* samples from Kioloa, including *Theileria* sp. AU_1048 (>99.6% 18S similarity) which was found in 22 *I. holocyclus* samples (13.12%, 95%CI = 8.4–19.1%), and a novel species designated *Theileria* sp. IH (for its putative vector *I. holocyclus*) that was identified in just three *I. holocyclus* samples from Kioloa and was most similar (94.12% 18S similarity) to *Theileria apogeana* (Figure S8, Table 2).

A third species, *Theileria* cf. *peramelis* was identified in three *I. holocyclus* samples and 10/11 (90.90%) *P. nasuta* samples from northern Sydney. *Theileria* 18S sequences were < 99% similar to, and form a monophyletic clade with *Theileria* cf. *peramelis* sequences from *P. nasuta*, and *I. tasmani* removed from *P. gunni* and *P. nasuta* hosts (Egan, Taylor, Austen, et al., 2021; Loh et al., 2018) (Figure S8).

### 3.6 *Trypanosoma* spp. in ticks in wildlife

*Trypanosoma* spp. (trypanosomes) contigs were one of the most abundant and prevalent taxa identified in tick and wildlife samples and were present in *H. bancrofti* samples from Kioloa (14/31 samples), *I. holocyclus* samples from Kioloa (19/99 samples) and northern Sydney (2/46 samples), *R. fuscipes* from Kioloa (5/11 samples, 45.5%) and one *T. vulpecula* sample from northern Sydney (1/3, 33.33%) (Table 2). Trypanosome contigs were found in 19 *I. holocyclus* (9.2%, 95%CI = 5.6–14.1%) samples from Kioloa (MRA: 12.4%) and three *I. holocyclus* (6.3%, 95%CI  = 1.6–16.4%) samples from northern Sydney (MRA:0.31%) (Table 2, Figure S4). These contigs were exclusively found in nymph samples and only one species, *T. gilletti*, was identified in all *I. holocyclus* samples (98.9–99.9% 18S similarity) (Figure S9).

*Haemaphysalis bancrofti* ticks from Kioloa contained trypanosome contigs in all life stages but were most abundance in nymphs (MRA: 9.74%) compared to males (MRA: 3.18%) or females (MRA 1.69%) (Figure S6). Analysis of 18S sequences identified two distinct *Trypanosoma* species in *H. bancrofti* samples, with *T. vegrandis* detected in four samples (4.8%, 95%CI = 1.5–11.2%) (< 98.1% 18S similarity), and a novel species designated *Trypanosoma* sp. HB (for its putative vector *H. bancrofti*) detected in 10 samples (12.4%, 95%CI = 6.2–21.7%) (Figure S9, Table 2). *Trypanosoma* sp. HB 18S sequence were most similar to other trypanosomes from Australian marsupials including *T. vegrandis*, *T. gilletti* and *T. copemani* (85.45%, 85.28% and 81.54% 18S similarity, respectively) and phylogenetically clustered with these species (Figure S9). Unsuccessful attempts were made to amplify *Trypanosoma* gGAPDH sequences for further phylogenetic characterisation using the protocols of Hamilton et al. (2004) and McInnes et al. (2009), and no gGAPDH contigs were found in the metatranscriptomic data.

*Rattus fuscipes* also contained trypanosome contigs (MRA: 12.09%) with PCR confirming the presence of trypanosomes in 5/11 (45.54%) *R. fuscipes* samples and identified the species as a *T. lewisi*‐like (subgenus *Herpetosoma*) species with greatest similarity (99.93%) to *Trypanosoma* sp. BR042 which was recently described from *R. rattus* from Sydney (Egan, Taylor, Austen, et al., 2020) (Figure S9). Additionally, one (33.3%) *T. vulpecula* sample from northern Sydney was dominated by trypanosome contigs (relative abundance: 41.27%), and 18S sequences from this sample were up to 98.9% similar to a group of poorly classified *T. cyclops*‐like trypanosomes which have previously been identified in Australian leeches and marsupials, including *T. vulpecula* (Egan, Ruiz‐Aravena, Austen, et al., 2020; Egan, Taylor, Austen, Banks, et al., 2021; Hall et al., 2021; Hamilton et al., 2005) (Figure S9).

### 3.7 Tick‐associated viruses

The complete or partial genomes of 12 viruses were identified in tick samples (Table 2). The virome of *I. holocyclus* has been interrogated previously (Chandra et al., 2021; Harvey et al., 2019; O'Brien et al., 2018), and seven of the eight viruses identified are described therein. These include North Shore virus (*Partitiviridae*), Shelly Headland virus (*Reoviridae*), Timbillica virus (*Phenuiviridae*), Shelly Beach virus (*Reoviridae*), Ingleside virus (*Virgaviridae*), *Ixodes holocyclus* Iflavirus (*Iflaviridae*) and Genoa virus (*Chuviridae*) (Table 2).

**FIGURE 6:**
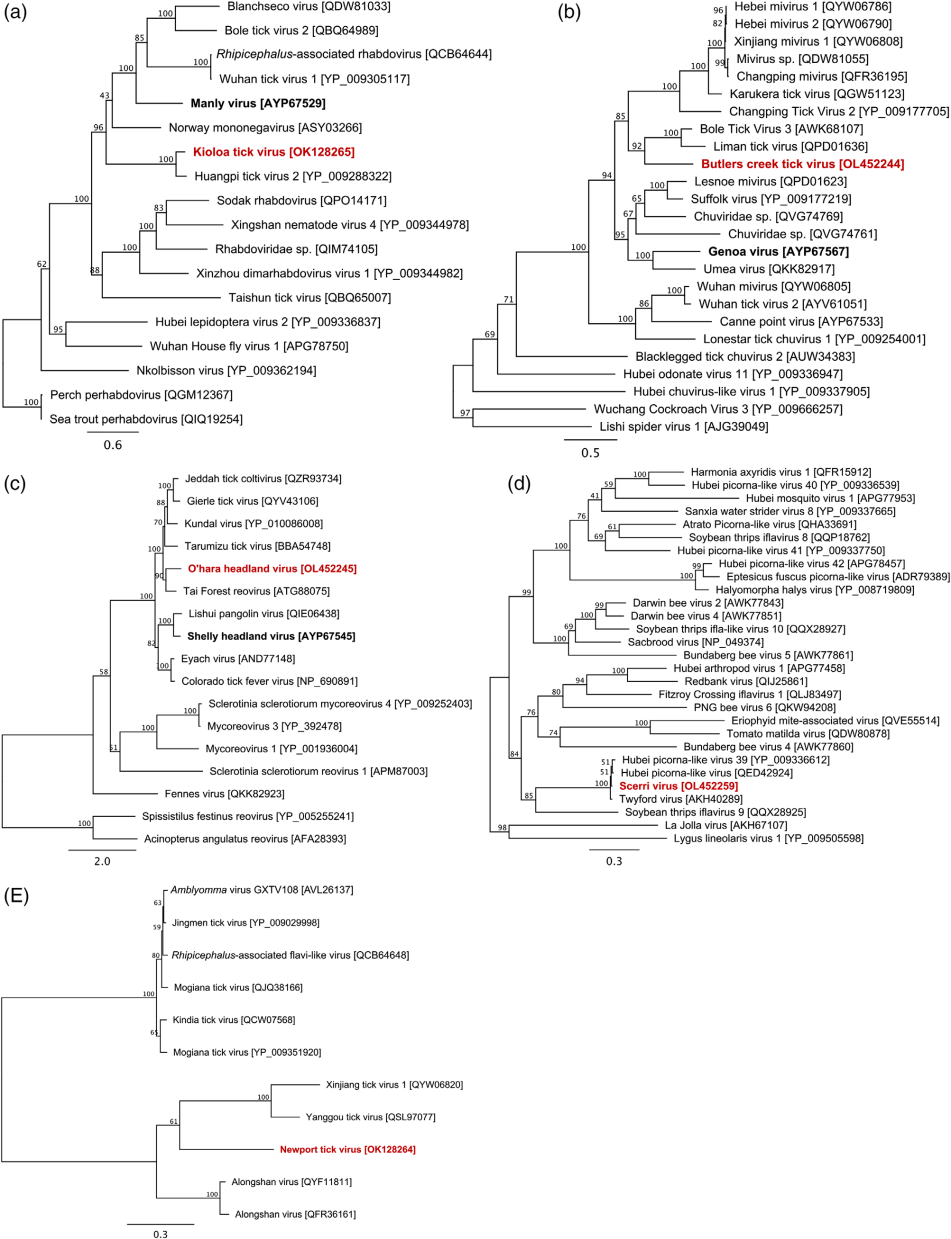
Maximum likelihood phylogenetic analysis of novel tick rhabdoviruses (2194 aa, RdRp) (a), chuviruses (437 aa, glycoprotein) (B), coltiviruses (1333 aa, RdRp) (c), iflaviruses (427 aa, ORF2) (d) and jingmenviruses (469 aa, VP1a) (e). Red bold text indicates sequences from this study, bold black text indicates previously describe Australian tick viruses. Square brackets indicate GenBank accessions

Novel *Flaviviridae*‐like contigs were identified in 41 *I. holocyclus* samples from Kioloa (24.4%, 95%CI = 17.6–32.7) and 25 *I. holocyclus* from northern Sydney (65.2%, 95%CI = 4.3–93.9%), and we have designated this virus as Newport tick virus (NTV) (Table 2). The largest NTV contigs (2087 nt) encoding a complete putative glycoprotein (VP1a) with > 70.2% nucleotide and > 62.4% amino acid (aa) homology to several recently described *Jingmenvirus* spp. (*Flaviviridae‐*like) including Alongshan virus, Yanggou tick virus and Jingmen tick virus. Several smaller NTV contigs (<300 bp) also had > 75.3% aa homology to the putative capsid protein and hypothetic protein of Alongshan virus. Phylogenetic analysis of the putative VP1a glycoprotein confirmed NTV clustered within the jingmenviruses and showed that NTV was distinct from other members of the group such as Alongshan virus and Yanggou virus (Figure 6).

The virome of *H. bancrofti* has not been investigated previously, and four novel viral genomes (one complete, three partial) were identified in *H. bancrofti* samples from Kioloa. The most abundant and prevalent virus was found in 23 *H. bancrofti* samples (44.4%, 95%CI = 28.1–66.9%) and belonged to a novel *Rhabdoviridae* sp. that was designated Kioloa tick virus (KTV) (Table 2). The whole genome (13,109 nt) of KTV was assembled and contained five complete genes in the standard N‐P‐M‐G‐L genome organisation of rhabdoviruses. Comparison of the RNA‐dependent RNA polymerase gene sequence showed that KTV was distinct from other rhabdoviruses but belonged to a monophyletic cluster of tick‐associated viruses within the dimarhabdovirus super group that includes Huangpi tick virus, Norway mononegavirus 1, Manly virus, Blanchesco virus and Wuhan tick virus 1 (82.4%, 47.1%, 45.2%, 42.6% and 42.2% aa similarity, respectively) (Figure 6).

In addition to the complete genome of KTV, three partial viral genomes were detected in *H. bancrofti* samples, including putative *Chuviridae*, *Reoviridae* and *Iflaviridae* spp. *Chuviridae* contigs (max: 1535 nt), designated Butlers creek virus (BCV) was identified in seven *H. bancrofti* samples (8.3%, 95%CI = 3.6–15.9%) and encode a partial (438 aa) putative glycoprotein which shares 64.5% aa homology with Bole tick virus 3 and phylogenetically grouped with a monophyletic clade of tick‐associated miviruses from *Rhipicephalus* spp. hosts (Figure 6). *Coltivirus* contigs, designated O'Hara Headland virus (OHV), were identified in a single *H. bancrofti* sample (1.1%, 95%CI = 0.4–4.8%). OHV contigs (max: 3999 nt) encoded a partial RNA‐dependent RNA polymerase gene (1333 aa), which had highest homology (65.1 % aa identity) to Tai Forest virus, and phylogenetically grouped within the coltivirus genus, which includes important human tick‐borne pathogens such as Colorado tick fever virus (55.7% aa identity) and Eyach virus (55.3% aa identity).

Contigs (531–2418 nt) belonging to an *Iflavirus*‐like virus were identified in 8/31 *H. bancrofti* samples (10.7%, 95%CI = 4.9–20.1%), and we have named this virus Scerri virus (SV). SV contigs encoded two distinct open reading frames that were most similar (up to 98.1% aa identity) to Twyford virus and several Hubei *picorna*‐like viruses from Dipteran hosts (Figure 6).

### 3.8 Rodent‐associated pathogens

In addition to the *Hepatozoon* spp. and *T. lewisi*‐like parasites discussed above, several other rodent‐associated pathogens were identified in *R. rattus* and *R. fuscipes*. Several *Bartonella* spp. were detected, including *B. queenslandensis, B. cooperplainsensis* and *B. rattaustraliani* (Table 2, Figure S10). Metatranscriptomic sequencing also identified *Mycoplasma haemomuris* in 19 (57.57%) *R*. *rattus*, and *Spironucleus muris, Neospora caninum* and *Sarcocystis* sp. (GenBank: MZ502182) in one (3.03%) *R. rattus* each. Four *R. rattus* also contained contigs of the helminth parasites *Angiostrongylus cantonensis* and *Strongyloides ratti*, both of which are common parasites of *R*. *rattus* and were apparent during necropsy. Novel *Orbivirus* (*Reoviridae*), *pegivirus* (*Flaviviridae*), *pestivirus* (*Flaviviridae*) and *Annelloviridae* contigs were also detected in *R. rattus* and *R*. *fuscipes* blood samples (Table 2), all of which phylogenetically cluster within monophyletic rodent‐associated viral lineages (Du et al., 2018; Firth et al., 2014; Nishiyama et al., 2014; Wu et al., 2018) (Figure S11).

## 4 DISCUSSION

In this study, we utilised metatranscriptomic sequencing to simultaneously interrogate the prokaryotic, eukaryotic and viral biomes of questing tick and wildlife blood samples to provide a comprehensive audit of potentially zoonotic tick‐borne microorganisms in two sites in coastal New South Wales (NSW), Australia. We identified a wide range of taxonomically diverse vertebrate‐infecting microorganism in questing ticks including several potentially zoonotic agents such as *Borrelia* sp., coltiviruses and jingmenviruses. In addition, our results add to the growing literature demonstrating that Australian ticks and their wildlife hosts harbour a unique and endemic microbial diversity that is largely distinct from the tick‐borne pathogens found on other continents and concurs with previous reports that found no evidence of *B. burgdorferi s.l*. or other northern hemisphere tick‐borne pathogens in Australian ticks or wildlife.

*Borrelia* spp. have previously been identified in Australian *Bothriocroton* spp. ticks and their wildlife hosts including echidnas (*Tachyglossus aculeatus*) and *B. concolor* (Loh et al., 2016); lace monitors (*Varanus varius*) and *B. undatum* (Panetta et al., 2017); and wombats (*Vombatus ursinus*) and *B. auruginans* (Beard et al., 2021). There is little evidence that these *Borrelia* spp. are transmitted by anthropophilic tick species (such as *I. holocyclus*), and it is probable that their host range is constrained by the strict host associations of their putative primary *Bothriocroton* spp. vectors. While all Australian *Borrelia* spp. have not been comprehensively characterised, all appear to be distantly related to *B. burgdorferi s.l*. and most group with the monophyletic reptile‐associated *Borrelia* clade, which includes subclades that correlate strongly with specific host associations (i.e., Serpentes, *Varanus* spp., Testudines, echidna) (Figures 5, S5).

More recently, a novel spirochete closely related to putative *Borrelia* sp. R57 was identified for the first time in Australia in *R. rattus* (Egan, Taylor, Banks, et al., 2021). This spirochete belongs to a unique rodent‐associated lineage that is likely globally dispersed by anthropogenic rodents (Egan, Taylor, Banks, et al., 2021; Fedorova et al., 2014; H. Gil et al., 2005). The absence of these spirochetes in *R. rattus* blood samples in this study could be explained by the apparent tissue‐specific tropism of these spirochetes (Egan, Taylor, Banks, et al., 2021; H. Gil et al., 2005).

*Borrelia* sp. HB is the first member of the hard tick‐borne relapsing fever (HTRF) clade identified in Australia, which includes the human pathogen *B. miyamotoi*, the bovine pathogen *B. theileri*, *B. lonestari* and a variety of novel species associated with *Haemaphysalis* and *Rhipicephalus* vectors. *Borrelia* sp. HB is most closely related to Asian HTRF *Borrelia* genotypes from *Haemaphysalis* spp. including *H. flava*, *H. formosensis*, *H. japonica*, *H. kitaokai*, *H. longicornis* and *H. megaspinosa* in Japan (Furuno et al., 2017; Lee et al., 2014), *H. hystricis* in Malaysia (Khoo et al., 2017), *H. longicornis* in China (Yang et al., 2018) and *Haemaphysalis* spp. in Laos (Taylor et al., 2016). These *Borrelia* sp. have also been associated with cervid hosts including Sika deer (*Cervus nippon*) in Japan and Père David's Deer (*Elaphurus davidianus*) in China, as well as wild boar (*Sus scrofa*) and racoons (*Procyon lotor*) in Japan (Furuno et al., 2017; Kumagai et al., 2018; Yang et al., 2018).

The vertebrate hosts of *Borrelia* sp. HB were not identified in this study, and *H. bancrofti* has a wide host range with the majority of records from Macropodidae spp., cattle (*Bos taurus* and *B. indicus*) and humans (Barker & Walker, 2014; Laan et al., 2011). While there is limited evidence that *H. bancrofti* frequently parasitizes deer or wild boars in Australia (Laan et al., 2011; McKenzie et al., 1985), comprehensive surveys of deer and wild boar ectoparasites have not been conducted in Australia, and the level of parasitism by *H. bancrofti* could be underestimated. Therefore, a broad range of potential hosts including native and introduced species should be considered in future studies aiming to identify the vertebrate hosts of *Borrelia* sp. HB. Given the well‐known pathogenic potential of other HTRF *Borrelia* spp. (Telford et al., 2015), this novel species warrants further ecological and epidemiologic investigation to better understand its potential pathogenic risks.

The detection here of *Rickettsia australis*, *Candidatus* Neoehrlichia australis, *Candidatus* Neoehrlichia arcana and *Anaplasma bovis* was not unexpected, as these endemic tick‐borne bacteria have been reported previously (Egan, Loh, et al., 2020; Gofton et al., 2016; Hussain‐Yusuf et al., 2020). *R. australis* is the only human pathogen identified in this study, with the prevalence in questing *I. holocyclus* estimated to be 1.4% (95%CI = 0.3–3.5%) in Kioloa and 2.1% (95%CI = 0.1–8.9%) in northern Sydney. This is lower than other studies from northern NSW and Queensland that found infections prevalence as high as 15.4% (Graves et al., 2016; Hussain‐Yusuf et al., 2020); however, disparities in environment and reservoir host populations likely account for these differences.

NTV and OHV were identified here for the first time and phylogenetically cluster within the *jingmenvirus* (*Flaviviridae*‐like) and *coltivirus* (*Reoviridae*) genera, respectively (Figure 6). Another novel *coltivirus*, Shelly Headland virus, was also recently described from a limited number of *I. holocyclus* from NSW and was also detected here in *I. holocyclus* samples at a much higher prevalence than OHV (Table 2) (Harvey et al., 2019). Jingmenviruses and coltiviruses infect both arthropod and vertebrate hosts and include several emerging tick‐borne human pathogens such as Colorado tick fever virus, Eyach virus, Jingmen tick virus and Alongshan virus that are associated with cryptic febrile illnesses (Attoui et al., 2005; Jia et al., 2019; Temmam et al., 2019; Wang et al., 2019). These novel viruses warrant additional genomic and epidemiological characterisation to understand their potential to cause human disease.

Conversely, it is unlikely that KTV and BCV are pathogenic to humans based on their evolutionary grouping with other viruses. Although Longdon et al. (2015) suggests that most dimarhabdoviruses, including the clade that contains KTV, are arboviruses, no tick‐associated dimarhabdoviruses have ever been found in vertebrate hosts, with the study also concluding that rhabdovirus host switching events between more distantly related hosts is rare. SV contigs found in *H. bancrofti* samples were highly similar to several Hubei *picorna*‐like viruses (Figure 6) that infect *Entomophthora muscae*, a behaviour‐manipulating fungal pathogen of dipterans (Coyle et al., 2018). Ticks are susceptible to entomopathogenic fungal pathogens such as *Metarhizium* spp. (Goettel et al., 2005), and it is probable that SPV is associated with such a fungus.

Protozoan haemoparasites are widespread in Australian mammals, and our analyses identified several species from wildlife and ticks that concur with previous records. These include the detection of *B. lohae* from *T. vulpecula* and *I. trichosuri*, *Theileria* cf. *peramelis* from *P. nasuta* and *I. holocyclus*, *Trypanosoma* cf. *lewisi* from *R. fuscipes*, *Trypanosoma* cf. *cyclops* from *T. vulpecula*, *Hepatozoon* spp. from *R. rattus* and *R. fuscpes* and *H. ewingii* from *H. bancrofti* ticks. The identification and host/vector association of these endemic haemoprotozoa has been recently discussed (Egan, Taylor, Austen, et al., 2020; Egan, Taylor, Austen, et al., 2021; Greay et al., 2018). In addition, this work identified three novel haemoprotozoan species from questing *H. bancrofti* ticks: *Hepatozoon* sp. HB, *Theileria* sp. HB and *Trypanosoma* sp. HB, all of which phylogenetically cluster within well‐defined lineages associated with Australian mammals and/or tick vectors (Barbosa et al., 2019; Greay et al., 2018; Krige, Thompson, Seidlitz, Keatley, Wayne, et al., 2021).

Four new vector associations were also identified here, including the finding of *Theileria* sp. AU10_48 and *T. gilletti* in *I. holocyclus* and *T. vegrandis* and *B. mackerrasorum* in *H. bancrofti*. Both *Theileria* sp. AU_1048 and *B. mackerrasorum* have been recorded only once previously, with *Theileria* sp. AU_1048 identified in *Ixodes* sp. and *Haemaphysalis* sp. engorged on *Macropus giganteus* and *M. rufogriseus* in coastal NSW (Storey‐Lewis et al., 2018), and *B. mackerrasorum* from a single *Haemaphysalis* sp. engorged on a horse (*Equus caballus*) in NSW (Greay et al., 2018). This work confirms the identity of their tick vectors, and although their vertebrate hosts were not identified, it is likely based on our results and previous reports (Storey‐Lewis et al., 2018) that the hosts of *B. mackerrasorum* and *Theileria* sp. AU_1048 may include medium–large‐sized macropods, which are common hosts of *I. holocyclus* and *H. bancrofti* and common at sampling sites, but were not sampled here.

Globally, terrestrial trypanosomes are predominantly transmitted by insect vectors, but emerging evidence indicates that some Australian trypanosomes are transmitted by ticks. In addition to reports of endemic trypanosomes from engorged ticks (Austen et al., 2011; Barbosa et al., 2017), trypanosomes including *T. gilletti* and *T. vegrandis* were recently identified in questing *Ixodes* and *Amblyomma* spp. nymph and adult ticks in Western Australia, demonstrating for the first time the potential transstadial transmission of trypanosomes in ticks (Krige, Thompson, Seidlitz, Keatley, Botero, et al., 2021, Krige, Thompson, Seidlitz, Keatley, Wayne, et al., 2021). Our finding of *T. gilletti*, *T. vegrandis* and *Trypanosoma* sp. HB in questing *I. holocyclus* and *H. bancrofti* nymphs and adults adds to this emerging body of work, which collectively suggests that the transstadial transmission of trypanosomes occurs in ecologically and taxonomically diverse ticks. Nevertheless, empirical transmission studies are yet to be conducted to prove the role of ticks in the transmission of trypanosomes.

While this study identified a diverse range of tick‐borne microorganisms in wildlife blood samples, examination of other tissues from wildlife samples may have yielded additional microbial taxa with tissue‐specific tropisms, as demonstrated above for novel rodent‐associated spirochetes. However, ethical requirements to release native animals after sampling precluded the taking of additional tissue samples. Serological examination of serum samples and the collection of tissues from deceased animals (e.g., road kill) could have aided in the detection of further pathogenic microorganisms and should be considered in future studies as a compliment to live trapping.

Although we attempted to conduct our field sampling in a comprehensive manner, unavoidable disruptions from bushfires and SARS‐CoV‐2 travel restrictions severely hampered our efforts to systematically survey the tick and wildlife fauna at field sites. For this reason, the samples collected during this study may not accurately represent the tick or wildlife populations at each field site (indeed, Macropodidae species which are common in Sydney and Kioloa, NSW were not sampled at all).

Nevertheless, even with modest sampling at few localities, we identified a remarkably abundant and diverse array of vertebrate‐infecting tick‐borne microorganisms, several of which may have zoonotic potential. This outcome is in agreement with recent studies investigating Australian ticks, and collectively, these works convincingly highlight both the richness of Australia's tick‐borne microflora and how under‐explored this area is, particularly in relation to zoonotic agents (Chandra et al., 2021; Egan, Loh, et al., 2020; Egan, Taylor, Austen, et al., 2021; Egan, Taylor, Banks, et al., 2021; Gofton et al., 2015; Greay et al., 2018; Harvey et al., 2019). We expect therefore that more extensive sampling of diverse ticks and wildlife across Australia would uncover additional diversity and further novel tick‐borne microorganisms with zoonotic potential.

The distinction of this study compared to others applying metatranscriptomic techniques to the exploration of tick‐borne microorganisms is that our taxonomically impartial analysis allowed the identification of bacterial, protozoan and viral microorganisms without prejudice. Typically, metatranscriptomic studies are limited to the exploration of viral taxa only, and although highly appropriate for such studies, this technology also offers opportunities for the exploration of other biomes, as demonstrated in this work and other recent studies (Batson et al., 2021; Marcelino et al., 2019; Ortiz‐Baez et al., 2020). By removing taxonomic restrictions on the analysis of metatranscriptomic data, researchers can more accuratly investigate and characterise entire microbial communities rather than individual biomes in isolation, leading to the a deeper understanding of the members present, their interactions, and how they may affect phenotypic traits.

Unbiased disease surveillance within a One Health framework can provide a holistic perspective of pathogen diversity at the individual, host or landscape scale, and is a fundamental tool that enables us to identify and respond to novel emerging pathogens. This work identified a range of tick‐borne bacteria, protozoa and viruses from questing anthropogenic ticks and wildlife blood samples including several potentially zoonotic taxa related to known agents of human and animal disease. These potentially zoonotic taxa, including *Borrelia* sp. HB, NTV and OHV, warrants further study to clarify their pathogenic potential. Although this work cannot implicate these novel tick‐borne microorganisms as causes of disease, it does broaden our understanding of the richness of potentially zoonotic microbes associated with *I. holocyclus* and *H. bancrofti* and offers a springboard for further research to quantify, and hopefully mitigate, their zoonotic risk before potential disease emergence.

## CONFLICT OF INTEREST

The author declares that there is no conflict of interest.

## ETHICS APPROVAL

The authors confirm that the ethical policies of the journal have been adhered to and the appropriate ethical review committee approval has been received. The Australian Code for the Care and Use of Animals for Scientific Purposes were followed.

## AUTHOR CONTRIBUTIONS

Alexander W. Gofton, Ina Smith and Kim R. Blasdell conceived the study, coordinated, and performed field and laboratory work. Field work was performed with the assistance of Casey Taylor, Michelle Michie, Emilie Roy‐Dufresne and Jacqueline Poldy. Laboratory work was performed with the assistance of Michelle Michie, Michael Dunn and Jian Wang. Alexander W. Gofton performed bioinformatic and phylogenetic analyses with input from Mary Tachedjian and Kim R. Blasdell and wrote the manuscript, with inputs from Ina Smith, Kim R. Blasdell, Casey Taylor and Peter B. Banks.

## Supporting information



Supporting Material

## Data Availability

The data that support the findings of this study are openly available in the NCBI Sequence Read Archive (accession PRJNA777641).
