# Supplementary material for: Metatranscriptomic profiling reveals diverse tick‐borne bacteria, protozoans and viruses in ticks and wildlife from Australia
Source: Transbound Emerg Dis. 2022 May 16;69(5):e2389–407. doi: 10.1111/tbed.14581 (PMC9790515; doi:10.1111/tbed.14581)
Supplement: Supplementary file 1 — Supporting Material [file TBED-69-e2389-s001.docx]

**Supplementary materials:**

*Transboundary and Emerging Diseases*

**Metatranscriptomic profiling reveals diverse tick-borne bacteria, protozoans, and viruses in questing ticks and wildlife from Australia**

Alexander W. Gofton^1*^, Kim Blasdell^2^, Casey Taylor^3^, Peter B. Banks^3^, Michelle Michie^1^, Emilie Roy-Dufresne^1^, Jackie Poldy^1^*,* Jian Wang^1^, Mike Dunn^2^, Mary Tachedjian^2^, and Ina Smith^1^

^1^CSIRO, Health and Biosecurity, Canberra, ACT, Australia

^2^CSIRO, Health and Biosecurity, Australian Centre for Disease Preparedness, Geelong, VIC, Australia

^3^School of Life and Environmental Sciences, University of Sydney, Sydney, NSW, Australia

^4^ The Royal (Dick) School of Veterinary Studies, University of Edinburgh

*Corresponding author: [alexander.gofton@csiro.au](mailto:alexander.gofton@csiro.au)

**GPS coordinates for specific sampling locations presented in Figure 1.**

a) Willinga Park (GPS: -35.515848, 150.393352)

b) Australian National University Kioloa Campus Shelly Point bushland (-GPS: 35.539669, 150.383602)

c) Bangalay sand forest (GPS: -35.545969, 150.382558)

d) Australian National University Kioloa Campus Butlers Point bushland (-GPS: 35.550982, 150.377057)

e) Scerri Drive bushland (GPS: -35.558317, 150.379205)

f) O’Hara headland (GPS: -35.560464, 150.383978)

g) McKay Reserve (GPS: -33.605063, 151.323253)

h) Angophora Reserve (GPS: -33.638606, 151.31.5664)

i) Porter Reserve (GPS: -33.648976, 151.321477)

j) Irrawong Reserve (GPS: -33.692403, 151.283709)

k) Lumeah Reserve (GPS: -33.706454, 151.272981).

**Table S1.** PCR primer and thermal cycling conditions used to detect and confirm tick-associated microorganisms in tick and wildlife samples.

| **Primers and probes** | **Primer sequence (5’-3’)** | **Amplicon size** | **References** | |
| --- | --- | --- | --- | --- |
| Anaplasmataceae spp. 16S rRNA nested PCR | | | | |
| Ec9 | TACCTTGTTACGACTT | 1.4 kb | (Gofton et al., 2016) | |
| Ec12A | TGATCCTGGCTCAGAACGAACG |  | (Gofton et al., 2016) | |
|  | Cycling: 95°C 3m, (95°C 40 s, 48°C 1m, 72°C 90s x 32), 72°C 5m, 4°C hold | | | |
| A17a | GCGGCAAGCCTCCCACAT | 1.2 kb | (Gofton et al., 2016) | |
| IS58-1345r | CACCAGCTTCGAGTTAAACC |  | (Gofton et al., 2016) | |
|  | Cycling: 95°C 3m, (95°C 40 s, 54°C 1m, 72°C 90s x 32), 72°C 5m, 4°C hold | | | |
| *Rickettsia* spotted fever group spp. *gltA* qPCR | | | | |
| CS-F | TCGCAAATGTTCACGGTACTTT | N/A | (Stenos et al., 2005) | |
| CS-R | TCGTGCATTTCTTTCCATTGTG |  | (Stenos et al., 2005) | |
| CS-Probe | TGCAATAGGAAGAACCGTAGGCTGGATG |  | (Stenos et al., 2005) | |
|  | Cycling: 50°C 3m, 95°C 3m, (95°C 20 s, 60°C 40s x 40) | | | |
| *Rickettsia* spp. *ompB* PCR | | | | |
| 120_2788 | AAACAATAATCAAGGTACTGT | 816 bp | (Thu et al., 2019) | |
| 120_3599 | TACTTCCGGTTACAGCAAAGT |  | (Thu et al., 2019) | |
|  | Cycling: 95°C 3m, (95°C 30 s, 48°C 30s, 72°C 60s x 32), 72°C 5m, 4°C hold | | | |
| *Rickettsia* spp. g*ltA* PCR | | | | |
| gltA_Fc | CGAACTTACCGCTATTAGAATG | 580 bp | (Thu et al., 2019) | |
| gltA_Rc | CTTTAAGAGCGATAGCTTCAAG |  | (Thu et al., 2019) | |
|  | Cycling: 95°C 3m, (95°C 30 s, 55°C 30s, 72°C 45s x 32), 72°C 5m, 4°C hold | | | |
| *Coxiella burnetii* IS1111a qPCR | | | | |
| IS1111aF | GTTTCATCCGCGGTGTTAAT | N/A | (Banazis et al., 2010) | |
| IS1111aR | TGCAAGAATACGGACTCACG |  | (Banazis et al., 2010) | |
| IS1111aProbe | CCCACCGCTTCGCTCGCTAA |  | (Banazis et al., 2010) | |
|  | Cycling: 50°C 3m, 95°C 3m, (95°C 20 s, 60°C 45s x 40) | | | |
| *Bartonella* spp. ITS PCR | | | | |
| URBarto1 | CTTCGTTTCTCTTTCTTCA | 1.5 kb | (Roux & Raoult, 1995) | |
| URBarto2 | CTTCTCTTCACAATTTCAAT |  | (Roux & Raoult, 1995) | |
|  | Cycling: 95°C 3m, (95°C 30 s, 50°C 30s, 72°C 90s x 35), 72°C 5m, 4°C hold | | | |
| *Borrelia* spp. *flaB* nested PCR | | | | |
| 280-F | GCAGTTCARTCAGGTAACGG | 407 bp | (Clark et al., 2013) | |
| flaB-RL | GCAATCATAGCCATTGCAGATTGT |  | (Barbour et al., 1996) | |
|  | Cycling: 95°C 3m, (95°C 30 s, 52°C 30s, 72°C 40s x 32), 72°C 5m, 4°C hold | | | |
| 737-F | GCATCAACTGTRGTTGTAACATTAACAGG | 360 bp | (Clark et al., 2013) | |
| flaB-LL | ACATATTCAGATGCAGACAGAGGT |  | (Barbour et al., 1996) | |
|  | Cycling: 95°C 3m, (95°C 30 s, 55°C 30s, 72°C 30s x 32), 72°C 5m, 4°C hold | | | |
| *Borrelia* spp. 16S rRNA nested PCR | | | | |
| Bor-16S-F | TGCGTCTTAAGCATGCAAGT | Primary PCR | (Loh et al., 2016) | |
| Bor-1360R | GTACAAGGCCCGAGAACGTA |  | (Loh et al., 2016) | |
|  | Cycling: 95°C 3m, (95°C 30 s, 51°C 40s, 72°C 2m x 32), 72°C 5m, 4°C hold | | | |
| Bor-27F | CATGCAAGTCAAACGGAATG | 1.2 kb | (Loh et al., 2016) | |
| Bor1232R | ACTGTTTCGCTTCGCTTTGT |  | (Loh et al., 2016) | |
|  | Cycling: 95°C 3m, (95°C 30 s, 51°C 40s, 72°C 2m x 32), 72°C 5m, 4°C hold | | | |
| *Hepatozoon* spp. 18S rRNA | | | | |
| HepF300 | GTTTCTGACCTATCAGCTTTCGACG | 600 bp | (Ujvari et al., 2004) | |
| HepR900 | CAAATCTAAGAATTTCACCTCTGAC |  | (Ujvari et al., 2004) | |
|  | Cycling: 95°C 3m, (95°C 30 s, 58°C 30s, 72°C 45s x 32), 72°C 5m, 4°C hold | | | |
| *Babesia* and *Theileria* spp. 18S rRNA nested PCR | | | | |
| BTF1 | GGCTCATTACAACAGTTATAG | Primary PCR | (Jefferies et al., 2007) | |
| BTR1 | CCCAAAGACTTTGATTTCTCTC |  | (Jefferies et al., 2007) | |
|  | Cycling: 95°C 3m, (95°C 30 s, 58°C 30s, 72°C 45s x 32), 72°C 5m, 4°C hold | | | |
| BTF2 | CCGTGCTAATTGTAGGGCTAATAC | 800 bp | (Jefferies et al., 2007) | |
| BTR2 | GGACTACGACGGTATCTGATCG |  | (Jefferies et al., 2007) | |
|  | Cycling: 95°C 3m, (95°C 30 s, 62°C 30s, 72°C 45s x 32), 72°C 5m, 4°C hold | | | |
| **Primers and probes** | **Primer sequence (5’-3’)** | **Amplicon size** | | **References** |
| *Trypanosoma* spp. 18S rRNA nested PCR | | | | |
| SLF | GCTTGTTTCAAGGACTTAGC | Primary PCR | (McInnes et al., 2009) | |
| S762 | GACTTTTGCTTCCTCTAATG |  | (McInnes et al., 2009) | |
|  | Cycling: 95°C 3m, (95°C 30 s, 52°C 30s, 72°C 2m x 32), 72°C 5m, 4°C hold | | | |
| S825F | ACCGTTTCGGCTTTTGTTGG | 900 bp | (Maslov et al., 1996) | |
| S662R | GACTACAATGGTCTCTAATC |  | (Maslov et al., 1996) | |
|  | Cycling: 95°C 3m, (95°C 30 s, 52°C 30s, 72°C 2m x 32), 72°C 5m, 4°C hold | | | |
| S823F | CGAACAACTGCCCTATCAGC | 1 kb | (Maslov et al., 1996) | |
| SLIR | ATTGTAGTGCGCGTGTC |  | (Maslov et al., 1996) | |
|  | Cycling: 95°C 3m, (95°C 30 s, 52°C 30s, 72°C 2m x 32), 72°C 5m, 4°C hold | | | |
| *Ehrlichia canis gltA* qPCR | | | | |
| gltA-For | TAGCAACTTTATGGGGGCCA | 146 bp | (Thomson et al., 2018) | |
| gltA-Rev | TGACCAAAACCCATTAGCCTC |  | (Thomson et al., 2018) | |
| gltA-Probe | AGTAACGTAAAGCAGTTTATTCAA |  | (Thomson et al., 2018) | |
|  | Cycling: 95°C 5m, (95°C 15s, 56°C 30s x 40) | | | |
| Kioloa Tick Virus *Rhabdovirus* PCR | | | | |
| HF3_F1 | CTGTSTGTGCAGCWGAYATTG | 502 bp | This study | |
| HF3_R2 | GKRTCAGCGTARATGCGKGTC |  | This study | |
|  | Cycling: 50°C 30m, 94°C 2m (94°C 15s, 52°C 30s, 68°C 30s x 40), 68°C 5m, 4°C hold | | | |
| Newport Tick Virus *Jingmenvirus* PCR | | | | |
| AL_F1 | GACCAGCTACTAAGGCGGAAT | 441 bp | This study | |
| AL_R2 | GAACATCGTGTACTTCCACAG |  | This study | |
|  | Cycling: 50°C 30m, 94°C 2m (94°C 15s, 50°C 30s, 68°C 30s x 40), 68°C 5m, 4°C hold | | | |
| O’Hara Headland Virus *Coltivirus* PCR | | | | |
| Colt_F | CATGAGCACTACCTGACAGAC |  | This study | |
| Colt_R2 | CACAGTCAAAGGCTCGTCATG |  | This study | |
|  | Cycling: 50°C 30m, 94°C 2m (94°C 15s, 52°C 30s, 68°C 30s x 40), 68°C 5m, 4°C hold | | | |

R=A/G, W=A/T, K=G/T, Y=C/T, S=C/G

**PCR conditions**

PCR and qPCRs were performed using KAPA Taq PCR Kits (KAPA Biosystems, South Africa) with final reactions containing 1.5-2.5 mM MgCl_2_, 0.8 mM dNTPs, 0.4 µM of each primer, 0.5 µM of probe (if used) and 0.5 U KAPA Taq polymerase. Viral RT-PCRs were performed using Superscript III One step RT-PCR system with Platinum Taq containing 3.2mM MgCl_2_, 0.4 mM dNTPs with final concentrations of 0.2 µM of each primer.

**Metagenomic sequencing of *Borrelia*sp. nov. HB**

Samples positive for *Borrelia* sp. by nested PCR (Table S3) were interrogated with metagenomic shotgun sequencing in an attempt to generate additional genomic data for *Borrelia* genome assembly and phylogenomic analysis. Up to 1 µg of DNA was used to generate sequencing libraries with the Illumina® DNA Prep Kit (Illumina, USA) which were sequenced on the NovaSeq™ 6000 platform using 300 cycle paired-end chemistry (150 bp paired-end reads). Raw reads were trimmed and quality-filtered using cutadapt v2.8 (Martin, 2011) to remove sequencing adapter and distal bases and trimmomatic v.038 (Bolger et al., 2014) to trim and remove low quality reads. Reads were then mapped to the genome of *Borrelia*sp. HM str. tHM16w (GenBank: NZ_AP024401) (Nakao et al., 2021) together with metatranscriptomic reads from the samples using minimap2 (Li, 2018) producing 211 contigs. Each contig was aligned to complete *Borrelia* reference genomes (Fig. S5), and then alignments analysed with iqtree (Minh et al., 2020) with model selection (Kalyaanamoorthy et al., 2017) and 1000 ultrafast bootstrap replicates (Hoang et al., 2018). Average nucleotide identity was measured between genome alignment using OrthoANIu (Uoon et al., 2017).

**Table S2.** Cumulative summary of metatranscriptomic sequencing and assembly statistics

| **Species** | **Life stage/sex** | **Individuals/pools** | **QC read-pairs** | **Contigs** | **N50**  **(bp)** | **N10**  **(bp)** |
| --- | --- | --- | --- | --- | --- | --- |
| **Kioloa, NSW** | | | | | |  |
| *I. holocyclus* | Nymph | 1,055/34 | 1,502,374,650 | 52,477,787 | 654 | 3,739 |
|  | Male | 60/6 | 880,006,434 | 7,416,622 | 452 | 1,708 |
|  | Female | 80/10 | 314,972,442 | 15,918,470 | 455 | 1,914 |
| *H. bancrofti* | Nymph | 535/14 | 604,209,403 | 23,448,761 | 445 | 2,215 |
|  | Male | 40/6 | 248,439,758 | 8,121,673 | 485 | 2,670 |
|  | Female | 20/3 | 58,229,175 | 1,965,079 | 397 | 2,082 |
| *R. fuscipes* | - | 5/- | 43,573,214 | 1,592,786 | 286 | 5,861 |
| *T. vulpecula* | - | 3/- | 200,703,815 | 5,290,772 | 408 | 874 |
| **Sydney, NSW** | | | | | |  |
| *I. holocyclus* | Nymph | 210/7 | 187,736,784 | 6,225,718 | 826 | 4,706 |
|  | Male | 55/11 | 657,504,830 | 14,449,371 | 534 | 2,537 |
|  | Female | 70/14 | 892,978,778 | 36,550,998 | 596 | 2,258 |
| *I. trichosuri* | Nymph | 30/1 | 4,143,980 | 251,129 | 348 | 1,166 |
| *H. bancrofti* | Female | 5/1 | 1,171,909 | 40,798 | 338 | 947 |
| *R. rattus* | - | 30/- | 11,369,683,628 | 499,363,493 | 601 | 1,869 |
| *P. nasuta* | - | 5/- | 308,857 | 11,100 | 250 | 658 |
| *T. vulpecula* | - | 2/- | 20,858,957 | 765,385 | 328 | 1,142 |

**Figure S1.** Alpha rarefaction of tick (A) and wildlife blood (B) samples performed with a step size of 100 on TPM-normalised contig abundances.


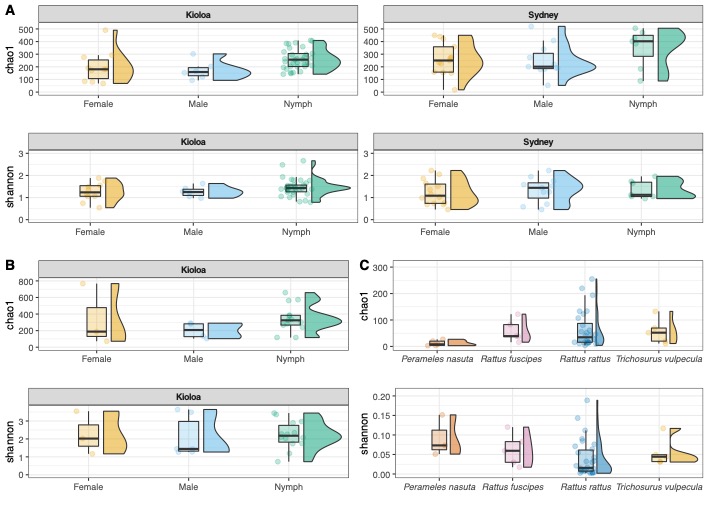


**Figure S2.** Box and violin plots of microbial alpha diversity levels in *Ixodes holocyclus* (A), *Haemaphysalis bancrofti* (B), and wildlife blood samples (C) using chao1 and Shannon indices on TPM-normalised contig abundances. Categories with only one sample (*Ixodes trichosuri* and *H. bancrofti* samples from Sydney) were excluded. No statistically significant (*p*<0.05) differences in the levels of microbial alpha diversity were detected between tick life stages between or within species, or between different wildlife species.

**Figure S3.** PCoA analysis of the microbial communities in *Ixodes holocyclus* (A), *Haemaphysalis bancrofti* (B) samples using Jensen-Shannon Divergences. Samples are divided by life stage and collection site with coloured polygons frame samples of the same category.

**Figure S4.** Heatmap of the relative abundance of the top 50 taxa in *Ixodes holocyclus* samples identified by metatranscriptomic analysis and summarised at the family level (or higher). Values represent the median relative abundance of TPM-adjusted contig counts.

**
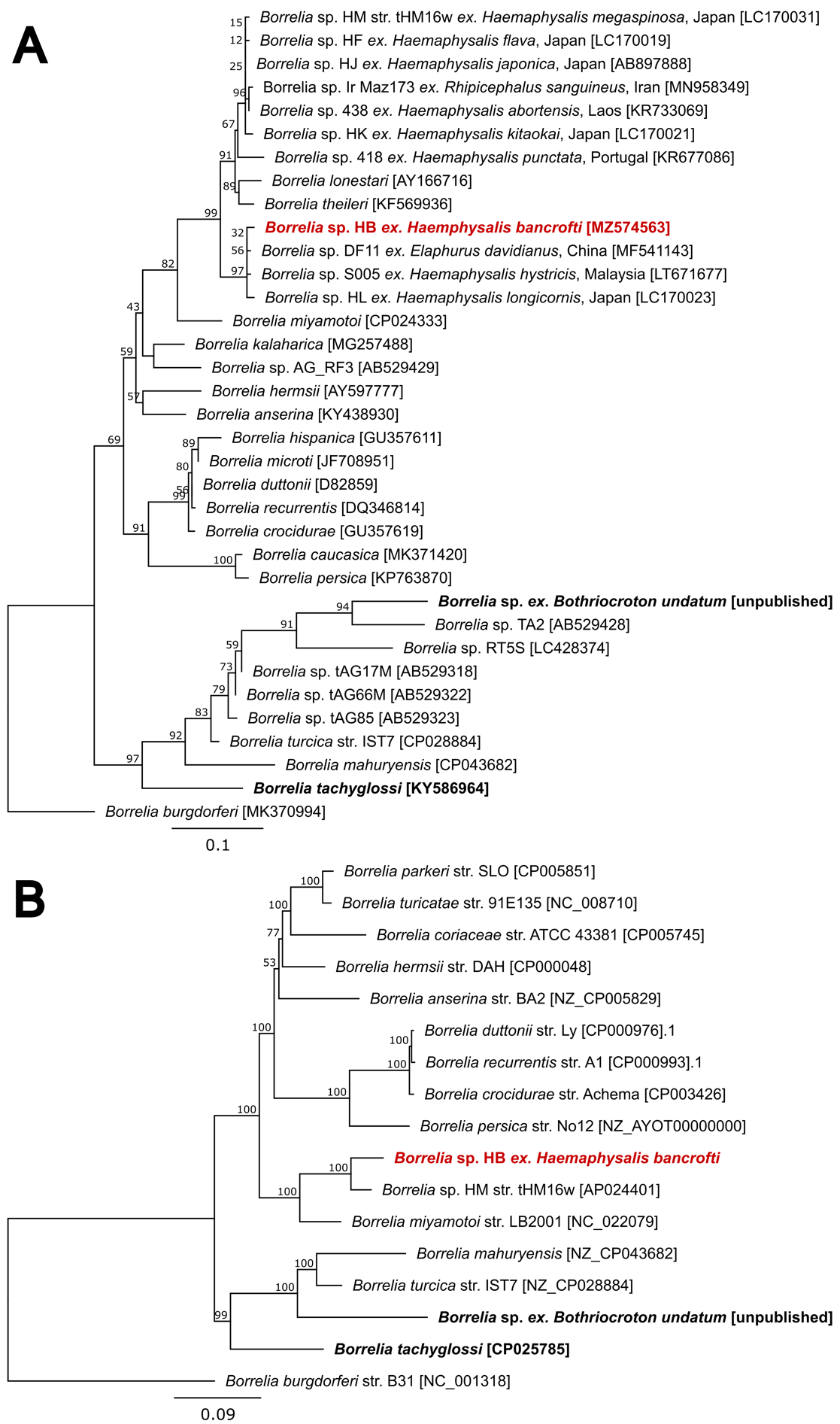
**

**Figure S5.** Phylogenetic analysis of *Borrelia* sp. HB based on 290 bp *flaB* sequences (A), and 45,510 bp of chromosomal contigs (B). Tree constructed using IQ-TREE with model selection and 5000 bootstrap replicates. Red bold text indicates sequences from this study, bold text indicates *Borrelia* spp. previously identified in Australia. Square brackets indicate GenBank accessions.

**Figure S6.** Heatmap of the relative abundance of the top 50 taxa in *Haemaphysalis bancrofti* samples identified by metatranscriptomic analysis and summarised at the family level (or higher). Values represent the median relative abundance of TPM-adjusted contig counts.

**
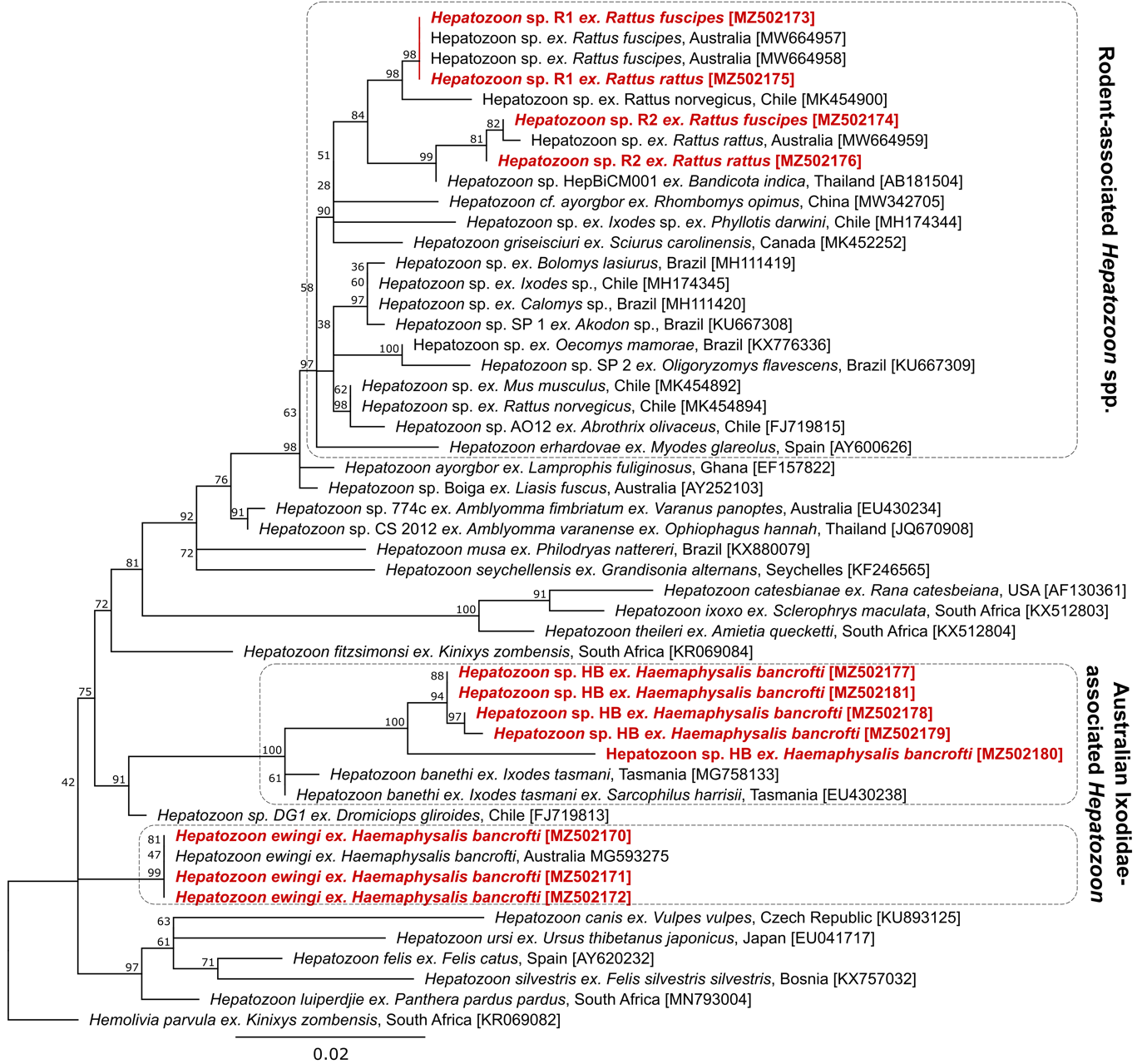
**

**Figure S7.** Maximum likelihood phylogenetic analysis of *Hepatozoon* spp. 18S rRNA sequences (585 bp). Tree constructed using IQ-TREE with model selection and 5000 bootstrap replicates. Red bold text indicates sequences from this study. Square brackets indicate GenBank accessions.

**
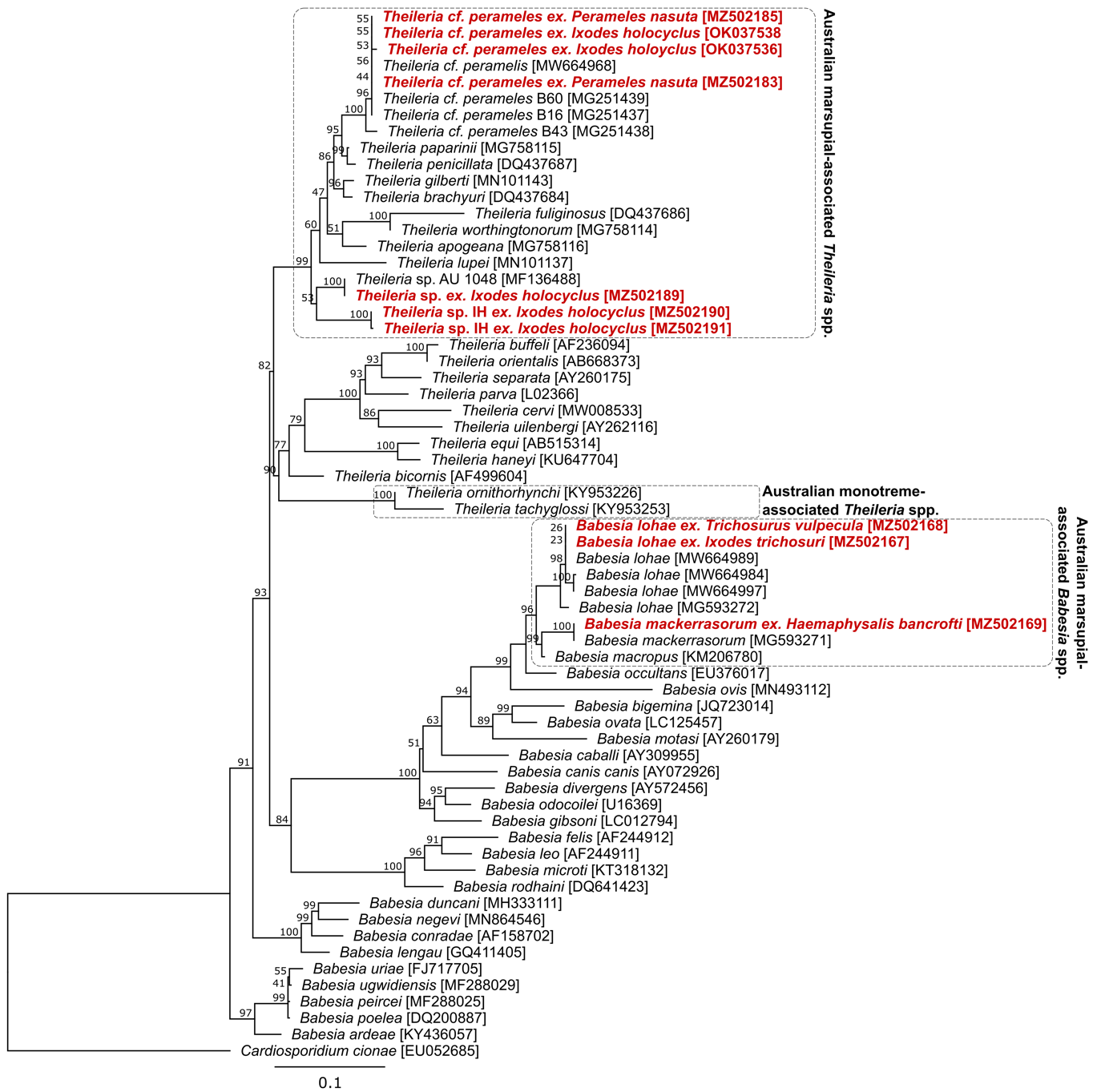
**

**Figure S8.** Maximum likelihood phylogenetic analysis of *Babesia* and *Theileria* spp. 18S rRNA sequences (850 bp). Tree constructed using IQ-TREE with model selection and 5000 bootstrap replicates. Red bold text indicates sequences from this study. Square brackets indicate GenBank accessions.

**
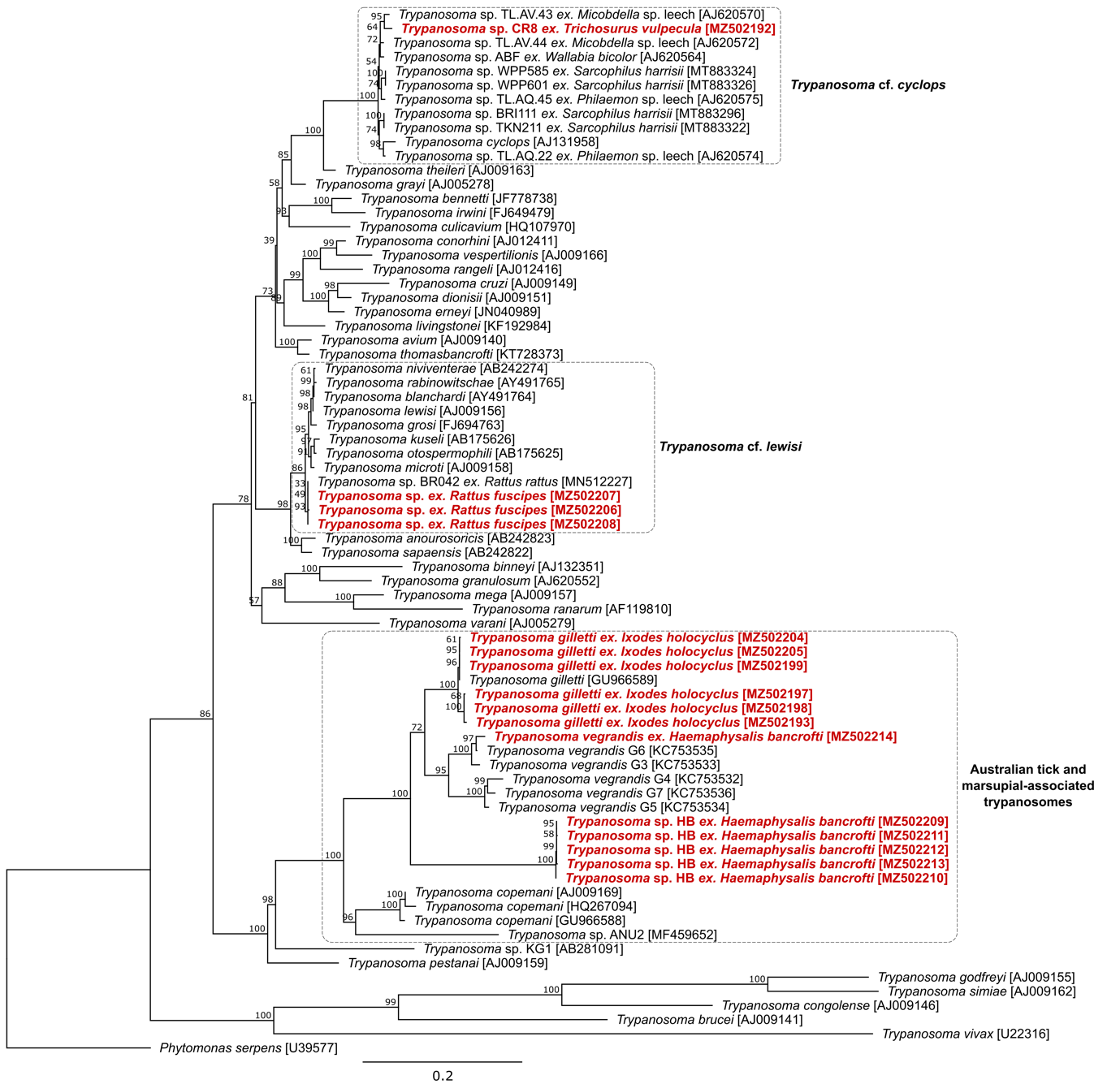
**

**Figure S9.** Maximum likelihood phylogenetic analysis of *Trypanosoma* spp. 18S rRNA sequences (1,500 bp). Tree constructed using IQ-TREE with model selection and 5000 bootstrap replicates. Red bold text indicates sequences from this study. Square brackets indicate GenBank accessions.


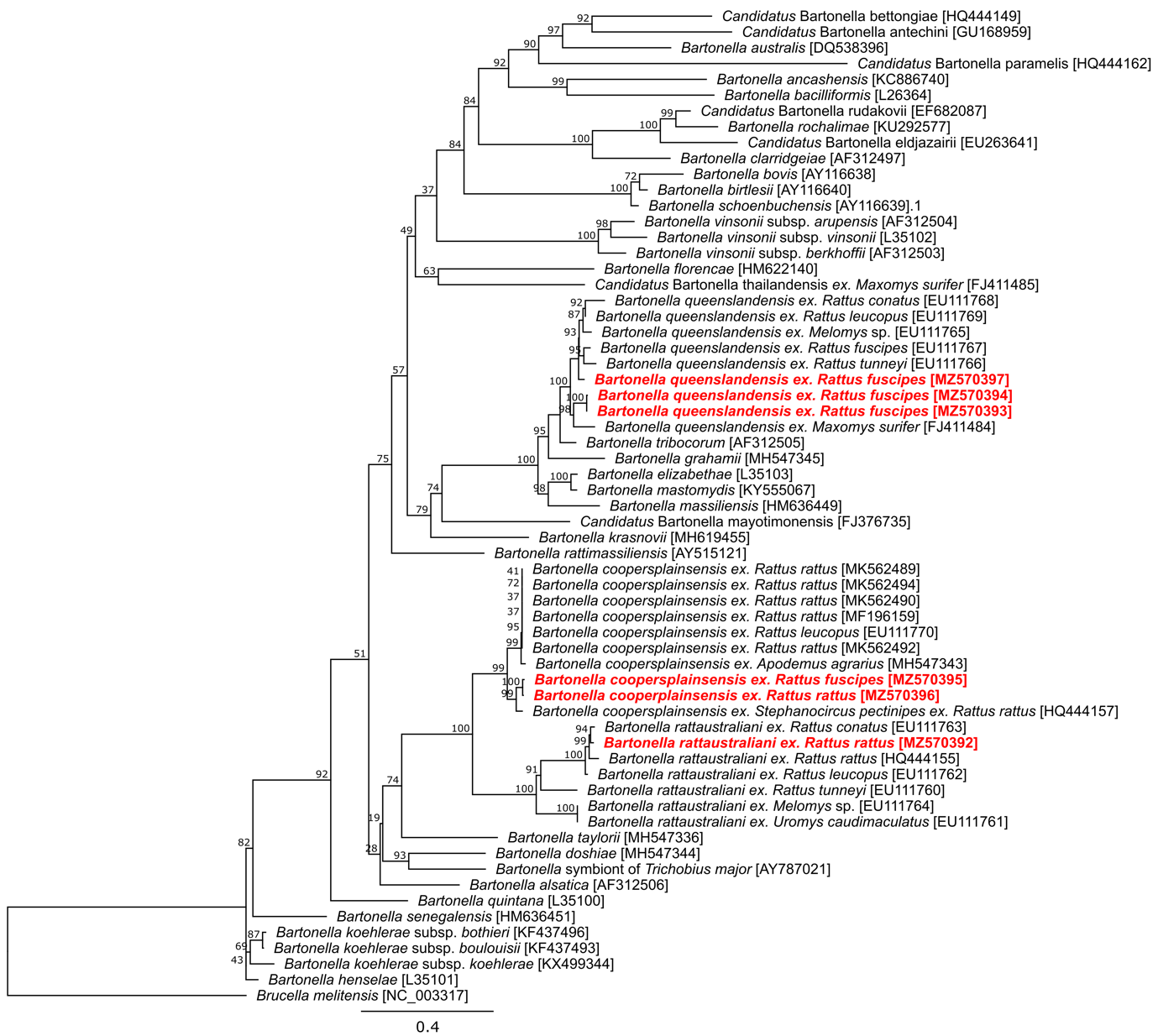


**Figure S10**. Phylogenetic analysis of *Bartonella* spp. internal-transcribed spacer sequences (616-777 bp). Tree constructed using IQ-TREE with model selection and 5000 bootstrap replicates. Red bold text indicates sequences from this study. Square brackets indicate GenBank accessions.


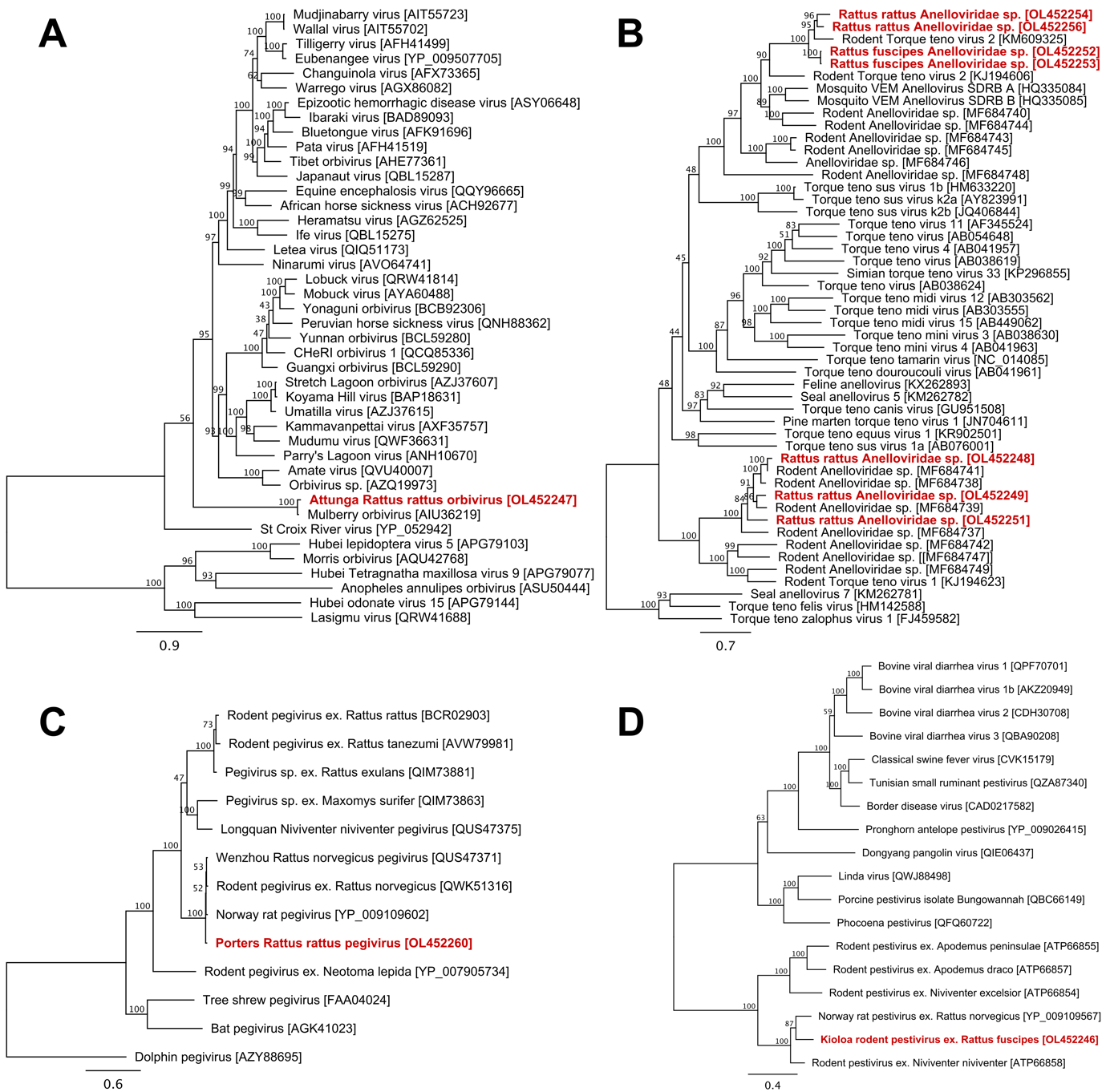


**Figure S11.** Maximum likelihood phylogenetic analysis of novel rodent orbiviruses (1,313 aa, RdRp) (A), anelloviruses (231-1,116 aa, ORF1) (B), pegiviruses (2,556 aa, polyprotein) (C), and pestiviruses (4,005 aa, polyprotein) (D). Trees constructed using IQ-TREE with model selection and 5000 bootstrap replicates. Red bold text indicates sequences from this study. Square brackets indicate GenBank accessions.

**References**

Banazis, M. J., Bestall, A. S., Reid, S. A., & Fenwick, S. G. (2010). A survey of Western Australian sheep, cattle and kangaroos to determine the prevalence of *Coxiella* *burnetii*. *Veterinary Microbiology*, *143*(2–4), 337–345. https://doi.org/10.1016/j.vetmic.2009.12.002

Barbour, A. G., Maupin, G. O., Teltow, G. J., Carter, C. J., & Piesman, J. (1996). Identification of an uncultivable *Borrelia* species in the hard tick *Amblyomma* *americanum*: Possible agent of a Lyme disease-like illness. *The Journal of Infectious Diseases*, *173*(2), 403–409. https://doi.org/10.1093/infdis/173.2.403

Clark, K. L., Leydet, B., & Hartman, S. (2013). Lyme Borreliosis in human patients in Florida and Georgia, USA. *International Journal of Medical Sciences*, *10*(7), 915–931. https://doi.org/10.7150/ijms.6273

Gofton, A. W., Doggett, S., Ratchford, A., Ryan, U., & Irwin, P. (2016). Phylogenetic characterisation of two novel Anaplasmataceae from Australian *Ixodes* *holocyclus* ticks: “*Candidatus* Neoehrlichia australis” and “*Candidatus* Neoehrlichia arcana.” *International Journal of Systematic and Evolutionary Microbiology*, *66*(10), 4256–4261. https://doi.org/10.1099/ijsem.0.001344

Jefferies, R., Ryan, U. M., & Irwin, P. J. (2007). PCR-RFLP for the detection and differentiation of the canine piroplasm species and its use with filter paper-based technologies. *Veterinary Parasitology*, *144*(1–2), 20–27. https://doi.org/10.1016/j.vetpar.2006.09.022

Loh, S.-M., Gofton, A. W., Lo, N., Gillett, A., Ryan, U. M., Irwin, P. J., & Oskam, C. L. (2016). Novel *Borrelia* species detected in echidna ticks, *Bothriocroton* *concolor*, in Australia. *Parasites & Vectors*, *9*(1), 339. https://doi.org/10.1186/s13071-016-1627-x

Maslov, D. A., Lukes, J., Jirku, M., & Simpson, L. (1996). Phylogeny of trypanosomes as inferred from the small and large subunit rRNAs: Implications for the evolution of parasitism in the trypanosomatid protozoa. *Molecular and Biochemical Parasitology*, *75*(2), 197–205. https://doi.org/10.1016/0166-6851(95)02526-x

McInnes, L. M., Gillett, A., Ryan, U. M., Austen, J., Campbell, R. S. F., Hanger, J., & Reid, S. A. (2009). *Trypanosoma* *irwini* n. sp (Sarcomastigophora: Trypanosomatidae) from the koala (*Phascolarctos* *cinereus*). *Parasitology*, *136*(8), 875–885. https://doi.org/10.1017/S0031182009006313

Roux, V., & Raoult, D. (1995). Inter- and intraspecies identification of *Bartonella* (Rochalimaea) species. *Journal of Clinical Microbiology*, *33*(6), 1573–1579.

Stenos, J., Graves, S. R., & Unsworth, N. B. (2005). A highly sensitive and specific real-time PCR assay for the detection of spotted fever and typhus group *Rickettsiae*. *The American Journal of Tropical Medicine and Hygiene*, *73*(6), 1083–1085.

Thomson, K., Yaaran, T., Belshaw, A., Curson, L., Tisi, L., Maurice, S., & Kiddle, G. (2018). A new TaqMan method for the reliable diagnosis of *Ehrlichia* spp. in canine whole blood. *Parasites & Vectors*, *11*(1), 350. https://doi.org/10.1186/s13071-018-2914-5

Thu, M. J., Qiu, Y., Matsuno, K., Kajihara, M., Mori-Kajihara, A., Omori, R., Monma, N., Chiba, K., Seto, J., Gokuden, M., Andoh, M., Oosako, H., Katakura, K., Takada, A., Sugimoto, C., Isoda, N., & Nakao, R. (2019). Diversity of spotted fever group rickettsiae and their association with host ticks in Japan. *Scientific Reports*, *9*(1), 1500. https://doi.org/10.1038/s41598-018-37836-5

Ujvari, B., Madsen, T., & Olsson, M. (2004). High prevalence of *Hepatozoon* spp. (Apicomplexa, Hepatozoidae) infection in water pythons (*Liasis* *fuscus*) from tropical Australia. *The Journal of Parasitology*, *90*(3), 670–672. https://doi.org/10.1645/GE-204R
